# Outsourcing health-care services to the private sector and treatable mortality rates in England, 2013–20: an observational study of NHS privatisation

**DOI:** 10.1016/S2468-2667(22)00133-5

**Published:** 2022-06-29

**Authors:** Benjamin Goodair, Aaron Reeves

**Affiliations:** a Department of Social Policy and Intervention, University of Oxford, Oxford, UK

## Abstract

**Background:**

The effects of outsourcing health services to for-profit providers are contested, with some arguing that introducing such providers will improve performance through additional competition while others worry that this will lead to cost cutting and poorer outcomes for patients. We aimed to examine this debate by empirically evaluating the impact of outsourced spending to private providers, following the 2012 Health and Social Care Act, on treatable mortality rates and the quality of health-care services in England.

**Methods:**

For this observational study, we used a novel database composed of parsable procurement contracts between April 1, 2013, and Feb 29, 2020 (n=645 674, value >£25 000, total value £204·1 billion), across 173 clinical commissioning groups (CCGs; regional health boards) in England. Data were compiled from 12 709 heterogenous expenditure files primarily scraped from commissioner websites with supplier names matched to registers identifying them as National Health Service (NHS) organisations, for-profit companies, or charities. We supplemented these data with rates of local mortality from causes that should be treatable by medical intervention, indicating the quality of health-care services. We used multivariate longitudinal regression models with fixed effects at the CCG level to analyse the association of for-profit outsourcing on treatable mortality rates in the following year. We used the average marginal effects to estimate total additional deaths attributable to changes in for-profit outsourcing. We provided alternative model specifications to test the robustness of our findings, match on background characteristics, examine the potential impact of measurement error, and adjust for possible confounding factors such as population demographics, total CCG expenditure, and local authority expenditure.

**Findings:**

We found that an annual increase of one percentage point of outsourcing to the private for-profit sector corresponded with an annual increase in treatable mortality of 0·38% (95% CI 0·22–0·55; p=0·0016) or 0·29 (95% CI 0·09–0·49; p=0·0041) deaths per 100 000 population in the following year. This finding was robust to matching on background characteristics, adjusting for possible confounding factors, and measurement error in our dataset. Changes to for-profit outsourcing since 2014 were associated with an additional 557 (95% CI 153–961) treatable deaths across the 173 CCGs.

**Interpretation:**

The privatisation of the NHS in England, through the outsourcing of services to for-profit companies, consistently increased in 2013–20. Private sector outsourcing corresponded with significantly increased rates of treatable mortality, potentially as a result of a decline in the quality of health-care services.

**Funding:**

Wellcome Trust.

## Introduction

In 2012, the Health and Social Care Act intensified pressures on the UK National Health Service (NHS) to outsource service provision from state-owned providers to private for-profit providers, but in doing so it created concerns that this would undermine the quality of care. In England, the NHS has long mixed private and public provision. Since 1991, its two-tier system, which consists of a private health sector serving a minority of the population and the NHS serving the majority, was blended with the introduction of an internal market for the NHS, constituting NHS purchasing bodies that contract services from a mixed pool of NHS-owned, for-profit, and non-profit providers, all of which serve NHS patients. Some services have remained predominantly delivered by NHS providers, but some services have been largely shifted towards a mixed market or mostly to independent providers. Facilities management and some ancillary services were quick to be contracted out to the private sector in the 1980s and 1990s, affecting the quality of these services.^1^, ^2^ Meanwhile, in the mid-2000s, reforms centred patient choice by introducing a consumer market that increased the use of private finance and independent sector treatment centres.^3^

The 2012 reforms deepened competition regulation, outlawing anti-competitive behaviour by commissioners with the aim of opening up the market so that more NHS services could be delivered by non-NHS providers;^4^, ^5^ this policy made it almost compulsory to outsource certain NHS services, or at least impossible to ensure contracts remained in the NHS.^6^, ^7^ The specifics of these reforms are outlined in secondary legislation, the “*Procurement, Patient Choice and Competition Regulations No. 2 (2013)*”,^8^ which directly ruled against any commissioning priority based on ownership status, meaning NHS providers could not be preferred over for-profit organisations by legal right.

**Research in context**

**Evidence before this study**

We searched Google Scholar, PubMed, and ProQuest for studies published in English from database inception up to Dec 1, 2021, assessing the relationship between health-care privatisation and health outcomes using the terms “healthcare privatisation”, “outsourcing”, “contracting”, “out”, “for-profit”, “healthcare quality”, “mortality”, and “NHS” applied to keywords, abstracts, and titles. We found various studies that largely compared outcomes between different health-care providers on the basis of ownership status. These studies had mixed findings regarding health-care quality and often concluded that different case-mixes of private and public hospitals make firm conclusions difficult. In the UK, studies commonly evaluate the aggregate health outcomes from differing levels of competition between providers, but have not accounted for levels of for-profit outsourcing. Evidence suggests mortality rates rose in Italy following a period of privatisation but whether the National Health Service (NHS) in England has even had substantial levels of privatisation is severely contested. To the best of our knowledge, no studies have been done to investigate the association of for-profit outsourcing at the NHS commissioner level with health outcomes in England.

**Added value of this study**

This observational study used a novel database with £204·1 billion of expenditure comprising 645 674 individual payments made by 173 NHS clinical commissioning groups in England between 2013 and 2020. We used these data to assess whether changes in the proportion of the expenditure being spent on for-profit companies are associated with changes in treatable mortality rates and, therefore, with the quality of health care. These data allowed us to conduct, to the best of our knowledge, the first empirical evaluation of a controversial health-care reform in England's recent history. Our findings help advance the debate about health-care privatisation considering that the extent of NHS privatisation in England was previously contested.

**Implications of all the available evidence**

Our study suggests that increased for-profit outsourcing from clinical commissioning groups in England might have adversely affected the quality of care delivered to patients and resulted in increased mortality rates. Our results provide the first assessment of creeping privatisation in England since controversial reforms were introduced in 2012 to encourage outsourcing of services, and our findings are corroborated in other contexts of health-care privatisation. Our findings suggest that further privatisation of the NHS might lead to worse population health outcomes.

The ensuing period of for-profit outsourcing from the NHS in England has coincided with worsening of some indicators of health-care quality. Treatable mortality rates have stagnated since 2013, breaking from a declining trend over the previous 10 years and leaving England with mortality rates that compare poorly with other high-income countries.^9^ Similarly, increases in waiting times and decreased patient satisfaction suggest the NHS is failing to maintain standards of care.^10^ Although austerity measures during this period have almost certainly played a role,^11^, ^12^ in this study we examined whether outsourcing to for-profit companies has contributed to this increase in treatable mortality.

This rise in treatable mortality potentially confirms the worries of those who were sceptical that outsourcing to independent health-care providers would incentivise providers to introduce innovative practices and improve overall performance.^13^ This has occurred in other countries, such as when mortality rates rose in Italy following a period of privatisation, and in other parts of the NHS in England, such as when cleaning services were outsourced.^14^, ^15^

Why might for-profit outsourcing be related to aggregate treatable mortality? One theory is that cost-cutting behaviours by for-profit providers mean that having more, and inferior, services provided by for-profit providers will lead to worse health-care quality and worse health outcomes.^16^ Another key dynamic is the different case-mixes often observed between for-profit and public providers—a result of so-called cream-skimming and concentrating the most complicated cases with public providers, which have no extra staff or funding to compensate.^17^

However, evidence of the impact of so-called creeping privatisation in general, and in the NHS in England in particular, remains uncertain. In general, findings are often inconclusive in that they do not analyse the aggregate effect of outsourcing on service-wide performance.^18^, ^19^ Moreover, comparisons between for-profit and public providers are often inappropriate because the case-mixes of private and public services are considerably different.

The 2012 reorganisation created new bodies for NHS health procurement, termed clinical commissioning groups (CCGs), which replaced the old primary care trusts, and responsibility for public health services was transferred to local authorities. CCGs were also individually required to publish their expenditure data, which produced discrepancies in the location and availability of commissioning expenditure data; these discrepancies made evaluation of outsourcing previously unfeasible.

We aimed to examine the impact on treatable mortality of increased outsourcing to private for-profit providers from CCGs in England during the period immediately following the implementation of the 2012 Health and Social Care Act.

## Methods

### Overview

The biggest challenge preventing evaluation of outsourcing from the NHS in England until now has been the absence of a harmonised data resource suitable for analysis. For this observational study, we used a novel database compiling parsable procurement expenditures between April 1, 2013, and Feb 29, 2020 (n=645 674, value >£25 000, total value £204·1 billion). This resource allowed us to analyse the impact of for-profit outsourcing in unprecedented detail, by conducting, to the best of our knowledge, the first robust empirical assessment of the impact of for-profit outsourcing from the NHS following the 2012 Health and Social Care Act on health outcomes.

### Data collection

Procurement expenditures were sourced from each CCG's website using web scraping tools. 12 709 data files containing CCG expenditures were downloaded, parsed, and cleaned. The names of suppliers in these files were then matched to names in the Companies House Register, central register of charities, and NHS Digital by algorithmic reconciliation of the names of suppliers. Full details of the curation process along with access to the underlying raw data are available from Rahal and Mohan.^20^ The method builds on recent progress to scrape, parse, and merge disaggregated public payments datasets, making them into accessible data resources with many applications in research and policy.^21^, ^22^

The response variable used in this study was our measure for health-care quality, treatable mortality, defined as “deaths that can be mainly avoided through timely and effective healthcare interventions, including secondary prevention and treatment”.^23^

According to the Office for National Statistics: “Treatable mortality measures the effectiveness of timely healthcare interventions, including secondary prevention and treatment.”^23^ This measure is an age-standardised rate of mortality per 100 000 population for specific causes of death—a full list of causes that are considered treatable is provided in the appendix (p 32). However, CCGs represent registered patients of general practitioners (GPs) through membership, rather than representing a geographical population. Consequently, our treatable mortality measure is an approximation of population outcomes in a given area rather than precise outcomes for patients using CCG services.

The explanatory variable of interest was a measure of outsourcing, defined as commissioning expenditure which is received by for-profit companies as a percentage of total expenditure. This definition excluded expenditure received by private non-profit organisations—all those registered to the central register of charities—as we specifically focused on the aggregate effects of outsourcing to providers that have profit-maximisation incentives.

Data were collected for all live CCGs in England as of 2019. Of the full sample of 191 CCGs, 173 provided at least some machine-readable data between 2013 and 2020, although most CCGs had years of data missing because of mergers or missing periods in data publication. Covariates in the model included annual total CCG spending, annual local authority spending per capita (total service expenditure), the claimant rate, population size, unemployment rate, average disposable household income, and proportions of the local population from minority ethnic backgrounds, with a degree-level qualification, or in a professional or managerial occupation (see the appendix [pp 28–32] for a full description of missing data, a table listing summary statistics for all study variables, and full locations of the data, as well as a discussion of the data limitations).

### Statistical analysis

We ran fixed-effects and first-differences regression models on the association between outsourcing and treatable mortality rates in 2013; these models controlled for all time-invariant confounders at the regional level. We also ran our fixed-effects model using covariate balancing with propensity scores based on treatable mortality rates at the beginning of the time-series and the total number of active GPs in each CCG. Covariate balancing is an advanced matching method that can weight values to balance the model, accounting for differences in observations according to their value of a continuous treatment variable—in this case, for-profit outsourcing.^24^ These analyses were all reported with cluster-robust standard errors with small-N adjustments.^25^ Finally, we did a multi-level random intercepts model, clustering mortality rates for local authorities within their geographically overlapping CCGs, allowing the intercept to vary for each cluster to see whether CCG outsourcing explains mortality rates in their relative local authorities. The full models are summarised in the appendix (p 3).

We also analysed the data with two alternative response variables: raw numbers of treatable deaths and preventable mortality. We used the average marginal effects from the raw numbers of treatable deaths to predict how many extra deaths were attributable to increases in outsourcing since 2013 and plot a trend line of mortality were outsourcing to have remained constant since 2014. To check whether our results showed a relationship between outsourcing and some alternative cause of health outcomes, such as changes in social determinants of health, we also ran our regressions on preventable mortality—mortality due to causes that we would expect public health interventions to prevent and that are not necessarily treatable by the primary, acute, or community health services funded by the CCGs (appendix p 13).

### Sensitivity analysis

Our analysis was run on novel data that were produced by web scraping and algorithmic matching of contracts published in non-uniform formats. Despite multiple manual data verification checks, it is probable that a small amount of error existed in our outsourcing observations. To check whether potential error in the contract data influenced our inferences, we synthetically replicated the effect of error on our findings. We ran the linear fixed-effects model 10 000 times but multiplied each observation for outsourcing by a random number with a specified minimum and maximum limit. We then repeated this analysis five times with different maximum error sizes, the largest of which was 50% (replicated by multiplying each value by a random number between 0·5 and 1·5), far larger than we would expect to exist in the data. We then plotted the density of the resulting coefficients for outsourcing in each regression, simulating how random error could have affected the findings.

To account for potential bias in the main result from the choice of covariates in the model, we present a specification curve in the appendix that is combined with the random error loops (pp 16–17). Finally, we sequentially dropped each CCG from our fixed-effects model to test whether any individual CCG was driving a substantial amount of the average effect size (appendix pp 14–15).

### Role of the funding source

The funder of this study had no influence on data collection, data analysis, data interpretation, writing of the manuscript, or the decision to submit for publication.

## Results

**Figure 1:**
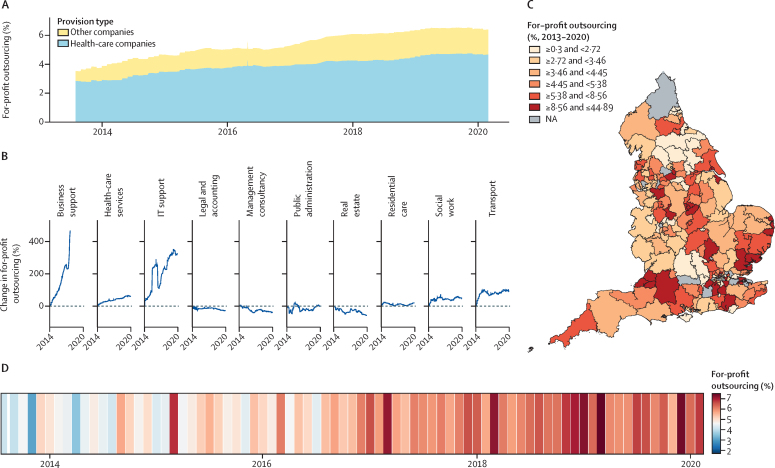
Levels of CCG outsourcing to for-profit organisations from 2013 to 2020 (A) Rolling percentage of total spending on health care and other for-profits. (B) Percentage change in total spending received by companies in different industrial sectors, based at zero for each sector's 2013–14 levels. (C) Total for-profit outsourcing over the entire time series for each CCG in England. (D) Levels of for-profit outsourcing across all CCGs each month. CCG=clinical commissioning group. NA=not available.

We found significant increases in for-profit outsourcing between 2013 and 2020 (appendix p 20). Figure 1 shows the changes in outsourcing since the beginning of April, 2013. Figure 1A shows a 365-day rolling average of total commissioning expenditure received by for-profit companies. It shows that overall levels of outsourcing to for-profit providers consistently increased since 2013, rising to more than 6% of total commissioner spending in England by 2020 (£323 million out of £4999 million for the first 3 months of 2020). It also shows that the majority of this outsourcing was received by health-care companies, defined as businesses with standard industrial classification divisions of human health activities.^26^

Figure 1B explores these classifications further, showing the percentage increase for the ten most highly procured industries. Relative to their outsourcing between 2013 and 2014, the largest increases were seen in spending on business support and IT support, with consistent increases in spending on health-care businesses, social work, and transport companies. Figure 1C shows the variation in the level of for-profit outsourcing by commissioner. For instance, east Berkshire CCG and Nottingham City CCG both spent £2·3 billion each on services between 2013 and 2020. However, east Berkshire spent around 2% on private companies, comprising a total of £46 million of outsourced contracts, while Nottingham City, with its heavy use of the CityCare partnership, outsourced more than 20%, aggregating to more than £450 million. Contrary to some claims, outsourcing from England's NHS commissioners to for-profit companies steadily increased since 2013, with a total of £11·5 billion of outsourced contracts received by for-profit companies between 2013 and 2020. Figure 1D represents the trends in privatisation of the NHS in England month by month; although levels of for-profit outsourcing varied on any given month, the increase between 2013 and 2020 was largely consistent.

The table shows the main results from our statistical analysis assessing the relationship between outsourcing and mortality rates.

We found in the fixed-effects model that an annual increase of one percentage point of outsourcing to the private sector was associated with an annual increase in treatable mortality of 0·38% (95% CI 0·22–0·55; p=0·0016) or 0·29 (0·09–0·49; p=0·0041) deaths per 100 000 population in the following year (see the appendix [p 5] for the model without log-transformed mortality rates calculating absolute effect size). In each model we found comparable effect sizes with significant, positive associations between increases in outsourcing and increases in treatable mortality in the following year.

The results from the covariate balancing models were robust to model specification, including full matching, choice of covariates, and removing any individual CCG from the data (see the appendix pp 14–20).

Since 2013, the annual numbers of treatable deaths in England has increased, breaking the trend of decreasing mortality for the previous 10 years. To calculate how much of the increase in treatable mortality could be explained by outsourcing, we did a fixed-effects regression analysis on the absolute number of treatable deaths, with the total spending on the private sector as the explanatory variable (appendix p 11). We found significant positive associations: an additional £1 million spent on for-profit companies corresponded with average increases of 0·29 (95% CI 0·05–0·53) deaths for all CCGs in the following year (p=0·0123). Between 2014 and 2019, there were total yearly increases of £927 million spent on for-profit providers by all 173 CCGs included in this study sample (appendix p 29). Based on the changes in for-profit spending and observed changes in treatable deaths for each CCG, we calculated that 557 (95% CI 153–961) additional deaths could have been attributed to changes in private-sector outsourcing between 2014 and 2019 across the 173 CCGs in the years for which we had data.

**Figure 2:**
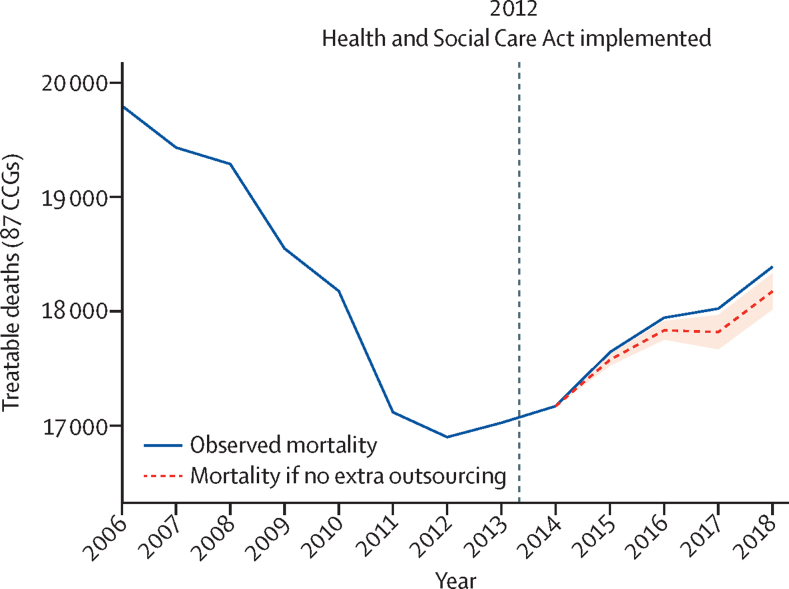
Treatable deaths from 2006 to 2018 The dashed grey line represents expected number of deaths if there had been no change to outsourcing since 2014. The shaded area represents the 95% CI. The expected trend line was constructed by subtracting the calculated additional deaths attributed to outsourcing for each CCG each year from the previous years’ synthetic death count by adding observed changes of the numbers of deaths. Data were trimmed to 2018 to maintain as many CCGs as possible as full observations of all variables were needed for each year. An updated version of this graph containing data up to 2019 is available in the appendix (p 12). CCG=clinical commissioning group.

Figure 2 shows the changes in total treatable deaths since 2008. For the 83 CCGs for which we had 5 years of consistent data, we plotted the observed total deaths before and after the introduction of the health-care reforms in April, 2013, and an expected trend had there been no change in outsourcing from 2014 onwards. Figure 2 shows that a considerable fraction of the increases in overall treatable mortality since 2013 can be associated with the outsourcing of services to the private sector.

We did two analyses to test whether the quality of health care might determine the relationship between for-profit outsourcing and treatable mortality. First, we assessed which types of outsourcing are associated with increases in mortality. We found that outsourcing to for-profit health-care companies was the only type of outsourcing associated with increasing mortality, suggesting that our results might be explained by the quality of health-care services delivered by these companies (appendix pp 6–8). An analysis treating preventable mortality as the response variable (appendix p 13) found no significant association between outsourcing and preventable mortality rates. Therefore, our findings suggest that our observed relationship between outsourcing and treatable mortality is not a product of general health outcomes in the population but is more directly associated with the quality of health-care services.

**Figure 3:**
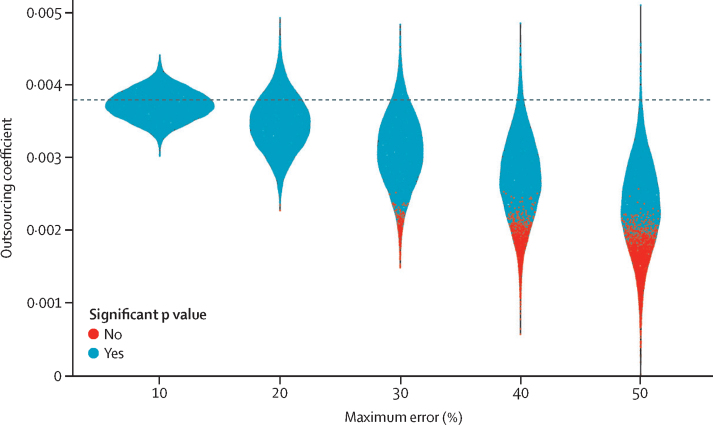
Synthetic random error The plot shows the density of the coefficient for outsourcing treatable mortality when running the regression 10 000 times with five different levels of random error. The horizontal dashed line represents the regression coefficient of for-profit outsourcing with observed values.

We also did a sensitivity analysis to account for any potential error in the contract data (figure 3). Figure 3 shows that if the outsourcing data contain random error up to 10% of the magnitude of the values, we could expect our effect size of outsourcing on treatable mortality to vary between 0·0030 and 0·0045. As the random error increased in magnitude we saw an expected shift in the modal coefficient size towards zero and a wider distribution of coefficient sizes. However, even given very large levels of random error in the data, our finding was still comparable to the results of the main analysis, using observed values of outsourcing, in that the association between for-profit outsourcing and treatable mortality was almost always positive and trends were close to our observed coefficient size of 0·0038 and, in the majority of cases, were significant.

We combined our random error simulation (figure 3) with a specification curve (appendix pp 16–18). All possible specifications reported similar findings, with positive associations between outsourcing and treatable mortality. Finally, we ran the linear fixed-effects regression (table 1, fixed-effects model) 173 times, removing a different CCG on each loop, to check whether any single CCG was primarily driving our overall result (appendix pp 14–15). We found that all regressions returned a significant, positive result, suggesting that our results were not considerably biased by any single CCG.

**Table:** Outsourcing and treatable mortality from multivariate longitudinal regression models

	**Fixed effects**		**First differences**		**Covariate balancing (model 1)**		**Covariate balancing (model 2)**		**Multi-level model**	
	Log-transformed treatable mortality (95% CI)	p value	Log-transformed treatable mortality (95% CI)	p value	Log-transformed treatable mortality (95% CI)	p value	Log-transformed treatable mortality (95% CI)	p value	Log-transformed treatable mortality (95% CI)	p value
For-profit outsourcing (%)	0·0038 (0·0022 to 0·0054)	0·0016	0·0046 (0·0030 to 0·0062)	0·0005	0·0037 (0·0019 to 0·0055)	0·0041	0·0039 (0·0021 to 0·0057)	0·0028	0·0026 (0·0003 to 0·0050)	0·0292
Local authority spending (£1000s per person)	0·0039 (−0·0364 to 0·0442)	0·8612	−0·0023 (−0·0499 to 0·0453)	0·9320	−0·0017 (−0·1252 to 0·1218)	0·9793	−0·0054 (−0·1263 to 0·1156)	0·9325	0·0202 (−0·0129 to 0·0533)	0·2311
Total CCG spending, millions (£)	0·0004 (−0·0009 to 0·0016)	0·5809	0·0008 (−0·0007 to 0·0022)	0·3162	0·0002 (−0·0011 to 0·0014)	0·8010	0·0000 (−0·0012 to 0·0011)	0·9378	−0·0001 (−0·0003 to 0·0001)	0·4807
Population size	0·4502 (−0·7243 to 1·6247)	0·4619	0·7529 (−0·9707 to 2·4764)	0·4026	0·5507 (−0·5896 to 1·6911)	0·3541	0·6969 (−0·3965 to 1·7903)	0·2240	0·0168 (−0·0176 to 0·0512)	0·3384
Average disposable household income	−0·1626 (−0·6383 to 0·3130)	0·5098	0·3481 (−0·1463 to 0·8424)	0·1821	−0·1546 (−0·7073 to 0·3981)	0·5871	−0·1042 (−0·6509 to 0·4424)	0·7108	−0·3634 (−0·4510 to −0·2758)	<0·0001
Numbers observed	609	..	450	..	517	..	553	..	534	..
*R*^2^	0·040	..	0·048	..	0·896	..	0·893	..	..	..
Conditional *R*^2^	..	..	..	..	..	..	..	..	0·813	..
Akaike information criterion	..	..	..	..	−1145·2	..	−1230·2	..	−962·6	..
Bayesian information criterion	..	..	..	..	−516·4	..	−552·7	..	−894·1	..
Intraclass correlation coefficient	..	..	..	..	..	..	..	..	0·3	..
Log likelihood function	..	..	..	..	720·576	..	772·087	..	..	..

The table shows the estimated annual increase in log-transformed treatable mortality rate against the same dependent variables using five different model specifications. Clinical commissioning group (CCG) fixed effects, time fixed effects, clustered standard errors, and demographic control variables were integrated into all models, with the exception of the multi-level model, which did not include CCG fixed effects. Numbers observed represent the number of observations used in each analysis. Numbers differ for each analysis due to missing data and model specifications. *R*^2^ and conditional *R*^2^ report the amount of variation in treatable mortality rates accounted for by the explanatory variables. The Akaike information criterion, Bayesian information criterion, and log likelihood function assess the goodness of fit of the models. The intraclass correlation coefficient shows how much of the variation in treatable mortality at the local authority level is explained by their CCG clusters.

* For-profit outsourcing, local authority spending, and CCG spending have a 1-year lag. Treatable mortality, population, and income are log transformed. Full model expressions are available in the appendix (p 4). Robust standard errors are clustered at the CCG level and use a bias-reduced linearisation estimator. Satterthwaite degrees of freedom are used to calculate estimates in the multi-level model. Demographic control variables included degree of education (%), managerial or professional occupation (%), ethnic minority (%), unemployment rate (%), and claimant rate (%).

## Discussion

The levels of outsourcing to for-profit health-care providers from NHS commissioners in England have increased considerably since 2013, rising to more than 6% of the total reported expenditure in 2020; £323 million of £4999 million went to for-profit companies in the first 3 months of 2020. The observed increase in for-profit outsourcing can resolve some of the debates and claims that there are not granular enough data to know whether the NHS in England has experienced a period of privatisation in recent years.^27^ Using a novel dataset based on procurement contracts for 173 CCGs, we found that increased outsourcing from CCGs in England was associated with an increase in mortality from treatable causes, potentially caused by worsening in the quality of health-care services.

Since the reforms to the NHS in England in 2012, some measures of health-care quality, as well as population health, have been worsening.^28^ Many have attributed these outcomes to austerity policies, leaving public services underfunded and having direct consequences on the social determinants of health through welfare cuts.^11^, ^12^ We suggest that outsourcing to for-profit companies is another way that the reforms of the post-financial crisis era have affected NHS service quality and mortality rates. However, with outsourcing being used as a mechanism for further austerity in some policy contexts, its relationship to health care deserves further attention.^29^ The marketisation of health-care services is underpinned by the beliefs that openness, competition, and management autonomy can improve the efficiency and performance of state-funded services.^30^ For decades, these principles have dictated the organisation of the NHS in England.^31^ However, our results suggest that these processes, manifesting in the outsourcing of health-care provision, are not associated with improvements in service provision, and instead have been associated with increased deaths among patients.

There are two primary ways that outsourcing to for-profit providers might lead to increased mortality. First, the private providers receiving NHS contracts could simply be delivering worse quality care, resulting in more health complications and deaths. For-profit providers tend to cut costs more than public providers; this can be through staff numbers and qualification levels or adherence to guidelines for correct medical processes.^18^ However, recent evidence finds no substantial difference in the rate of deaths from surgeries in private and public hospitals in England, even if selection effects make this estimation difficult.^32^ NHS surgeries might be delivered under more stringent conditions by for-profit companies than by NHS providers; however, differences in health outcomes are yet to be observed for those treated by NHS providers versus those treated by for-profit providers.

A second reason for the increased mortality rates could be that outsourcing leads to intensified pressure across the whole health system. Outsourcing can increase pressure on the wider system if profitable patients and services are cream-skimmed (ie, preferentially selected) by for-profit providers, creating a concentration of difficult treatments in public providers, as was witnessed in the NHS outsourcing to private hospitals during the 2000s.^17^ Similarly, increased competition for contracts could result in health-care providers prioritising easily quantified outcomes such as waiting times at the expense of quality of care, resulting in higher patient mortality, as was identified in the NHS after the pro-market reforms during the 1990s.^33^ The fact that we focused on a measure of health-care service performance and found no association between mortality and outsourcing when using a measure of mortality from causes that are treated by public health interventions suggests the overarching explanation for the increased mortality rates might be an aggregate decline in the quality of care. At the same time, more research is needed to unpack the precise mechanisms of worsening care in England since 2013, including an assessment of how private providers contribute to quality and safety data and systems of accountability. Another future avenue of research is the impact of outsourcing on health inequalities at the neighbourhood level, and the qualitative impact of access to health care.

These results have implications for the NHS privatisation debate, suggesting that for-profit provision of health-care services could be associated with worse population health outcomes. In the case of the NHS in England, our research raises doubts about whether the current extent of private sector use is optimal for the quality of care and suggests that further increases in for-profit provision would be a mistake. However, given the trends in the data, a change in direction and expansion of public sector provision seems unlikely without considerable political intervention.

The findings of this research are timely as new commissioning structures (integrated care boards) are about to replace CCGs entirely and, as in 2013, redraw the NHS market. This is a moment where once again the role of the private sector within the NHS in England must be scrutinised. The current analysis is also important given that, with only 42 integrated care boards replacing CCGs, such an analysis will not be possible in the future as local variation and accountability will be lost.

Limitations of this study include the length of time the data were available for, given the creation of CCGs in 2013, considerable mergers made at the beginning of 2020, and no legal requirement for their predecessors to publish expenditure data, which limited our ability to precisely measure outsourcing before 2013 or conduct before-and-after analyses. The associational nature of our findings cannot rule out the possibility of residual confounding, so our findings should not be interpreted as necessarily showing a causal relationship between outsourcing and mortality rates. Moreover, the expenditure data do not contain information about the specific services provided by the supplier; as such, further research is needed to establish whether some acute services are primarily responsible for the relationship between outsourcing to for-profit providers and increased mortality rates.

Since the passing of the 2012 Health and Social Care Act in England, for-profit companies are providing an increasing share of NHS services. Concerns about the quality of care provided by for-profit companies appear to be justified as our findings show that outsourcing is associated with higher rates of mortality from causes that could be treated by effective medical interventions.

## Data sharing

The extensive code library that accompanies this work can be found online. We used R (version 4.1.1) for all our analyses. The data that support the findings of this study, including replication materials, are all publicly available online. Raw data are provided in the appendix (p 30). CCG expenditure data are available from Rahal and Mohan.^20^

## Declaration of interests

We declare no competing interests.
