# Supplementary material for: Outsourcing health-care services to the private sector and treatable mortality rates in England, 2013–20: an observational study of NHS privatisation
Source: Lancet Public Health. 2022 Jun 29;7(7):e638–46. doi: 10.1016/S2468-2667(22)00133-5 (PMC10932752; doi:10.1016/S2468-2667(22)00133-5)
Supplement: Supplementary appendix [file mmc1.pdf]

THE LANCET
Public Health

Supplementary appendix

This appendix formed part of the original submission and has been peer reviewed. We post it as supplied by the authors.

Supplement to: Goodair B, Reeves A. Outsourcing health-care services to the private sector and treatable mortality rates in England, 2013–20: an observational study of NHS privatisation. *Lancet Public Health* 2022; **7:** e638–46.

Supplementary Material

Contents

Main Findings 3
S.1. Full Models of Main Finding 3
S.2 Full Model Specifications 4
S.3. Models without log transformations of Treatable Mortality 5
S.4. Standard Industrial Class 6
S.4.1 Division Groupings 6
S.4.2 Classes 7
S.4.3 Removing General Medical Practices 8
S.5 Time Fixed Effects 9
S.6. Nonparametric Covariate Balancing using Propensity Scores 10
S.7. Regression on absolute numbers of deaths 11
S.8 Additional deaths plot 12
Sensitivity Checks 13
S.9. Falsification test 13
S.10. Dropping individual CCGs 14
S.10.1 Plotting results 14
S.10.2. Full Model removing East Staffordshire 15
S.11. Specification curve 16
S.12. Combined Specification curve and random error simulation 17
S.12.1. Plotting all specification coefficients together 17
S.12.2. Selected specifications 18
S.13. Matching 19
Additional Analyses 20
S.14. Quadratic term 20
S.14. Growth curve models 20
S.14.1 Unconditional growth model 20
S.14.2 Conditional growth model 21
S.15 Difference in Difference 21
S.15.1 DiD Table 22
S.15.2 DiD graph 22
S.16. Interactions terms 23
S.16.1 Interaction with austerity 23
S.16.2 Interaction with demographics 24

S.16.3 Interaction between deprived and affluent CCGs ... 25
S.17. Spatial autocorrelation ... 26
S.18. Zoomed in maps ... 27
S.19. Ethnicity breakdown ... 27
Data ... 28
S.20. Data Cleaning ... 28
S.21. Missing Data ... 28
S.21.1 Missing Data of CCG Expenditure and Mortality ... 29
S.22. Model Data Summary ... 30
S.23. Data Locations ... 30
S.24. Causes of Treatable Mortality ... 32
S.25. CCG Level data ... 34
S.25.1 Table of Outsourcing and Treatable Mortality ... 34
S.25.2 Stripe plot ... 36
Reporting Checklists and Statements ... 37
S.26. STROBE Checklist ... 37
S.27. GATHER Checklist ... 40
S.28. Study protocol and data analysis plan statements ... 42

Main Findings

S.1. Full Models of Main Finding

Our main findings regress levels of outsourcing on the age standardised rate of treatable mortality. Here we present out full findings with an additional decimal place.

	ln(T. Mortality)				
	**FE**	**FD**	**CBPS (1)**	**CBPS (2)**	**MLM**
For-profit Outsourcing (%)	0.00382***	0.00458**	0.00372**	0.00390***	0.00265*
	(0.00083)	(0.00082)	(0.00092)	(0.00090)	(0.00121)
	[0.00163, 0.00601]	[0.00184, 0.00733]	[0.00142, 0.00602]	[0.00162, 0.00618]	[0.00027, 0.00503]
LA Spend (£000s per person)	0.00390	-0.00231	-0.00168	-0.00535	0.02022
	(0.02058)	(0.02427)	(0.06302)	(0.06169)	(0.01689)
	[-0.05147, 0.05927]	[-0.05996, 0.05534]	[-0.09739, 0.09404]	[-0.09991, 0.08921]	[-0.01288, 0.05333]
Total CCG Spend (£Ms)	0.00036	0.00077	0.00016	-0.00005	-0.00008
	(0.00065)	(0.00074)	(0.00063)	(0.00059)	(0.00012)
	[-0.00070, 0.00142]	[-0.00050, 0.00203]	[-0.00107, 0.00139]	[-0.00119, 0.00109]	[-0.00031, 0.00015]
Claimant Rate (%)	0.01312	-0.00004	0.01168	0.00981	0.10356***
	(0.01768)	(0.01731)	(0.01756)	(0.01709)	(0.01008)
	[-0.01507, 0.04131]	[-0.02453, 0.02446]	[-0.01806, 0.04143]	[-0.01919, 0.03880]	[0.08380, 0.12333]
Population size	0.45023	0.75287	0.55075	0.69691	0.01680
	(0.59924)	(0.87935)	(0.58182)	(0.55785)	(0.01755)
	[-0.61794, 1.51839]	[-1.09082, 2.59656]	[-0.57719, 1.67869]	[-0.39847, 1.79230]	[-0.01760, 0.05120]
Unemployment Rate (%)	0.00236	0.00429	0.00227	0.00229	-0.00216
	(0.00317)	(0.00363)	(0.00323)	(0.00315)	(0.00306)
	[-0.00352, 0.00825]	[-0.00164, 0.01022]	[-0.00384, 0.00838]	[-0.00370, 0.00828]	[-0.00815, 0.00383]
Ethnic Minority (%)	0.00204	0.00352	0.00107	0.00135	0.00773***
	(0.00207)	(0.00187)	(0.00206)	(0.00202)	(0.00084)
	[-0.00221, 0.00628]	[-0.00081, 0.00785]	[-0.00340, 0.00555]	[-0.00301, 0.00571]	[0.00609, 0.00937]
Degree Education (%)	-0.00047	-0.00140	-0.00005	0.00000	-0.00314***
	(0.00160)	(0.00163)	(0.00164)	(0.00148)	(0.00093)
	[-0.00345, 0.00250]	[-0.00470, 0.00191]	[-0.00329, 0.00320]	[-0.00304, 0.00305]	[-0.00496, -0.00133]
Average Disposable H.hold Income	-0.16263	0.34805	-0.15460	-0.10422	-0.36340***
	(0.24267)	(0.25223)	(0.28198)	(0.27891)	(0.04471)
	[-0.70864, 0.38338]	[-0.08845, 0.78455]	[-0.78957, 0.48037]	[-0.72355, 0.51512]	[-0.45104, -0.27576]
Managerial/Professional occupation (%)	-0.00224	-0.00164	-0.00111	-0.00125	-0.00073
	(0.00183)	(0.00176)	(0.00157)	(0.00150)	(0.00113)
	[-0.00553, 0.00105]	[-0.00505, 0.00177]	[-0.00473, 0.00250]	[-0.00470, 0.00221]	[-0.00294, 0.00147]
SD (Observations)					0.28188
Num.Obs.	609	450	517	553	534
R2	0.040	0.048	0.896	0.893	
R2 Adj.	-0.342	0.026	0.854	0.852	
R2 Marg.					0.717
R2 Cond.					0.813
AIC			-1145.2	-1230.2	-962.6
BIC			-516.4	-552.7	-894.1
ICC					0.3
Log.Lik.			720.576	772.087	
RMSE					0.08
CCG Fixed Effects	Yes	Yes	Yes	Yes	No
Time Fixed Effects	Yes	Yes	Yes	Yes	Yes
Clustered Standard Errors	Yes	Yes	Yes	Yes	Yes

+ p < 0.1, * p < 0.05, ** p < 0.01, *** p < 0.001
Outsourcing, LA Spend, and CCG Spend have a one-year lag.
Tr. Mortality, Population and GDHI are log transformed, 'Ln' denotes the natural log of outcome variable.
For full model expressions see supplementary material (sX)
Robust SEs are clustered at individual level and use a bias-reduced linearization estimator (CR2)
Satterthwaite degrees of freedom used in MLM

An ICC of 0.3 suggests a low level of the variation in LA mortality rates is explained by clustering at the CCG level. Given the imperfect nature of overlapping CCG and LA boundaries a low ICC may be expected.

S.2 Full Model Specifications

Our primary model 1 is a fixed effects model with fixed effects for year and CCG. The model is:

$$MORT_{it} = \beta OUT_{it} + \beta LASPEND_{it} + \beta CCGSPEND_{it} + \beta CLAIM_{it} + \beta POP_{it} + \beta UNEMP_{it} + \beta ETHMIN_{it} + \beta DEG_{it} + \beta INC_{it} + \beta OCC_{it} + \alpha_i + \mu_{it}$$

Where i is a given CCG, t is a given year, MORT is treatable mortality rate, OUT is for-profit outsourcing, LASPEND is Local Authority expenditure per person, CCGSPEND is total CCG expenditure, CLAIM is the claimant rate, POP is the CCG rough geographic area population, UNEMP is the unemployment rate, ETHMIN is the percent of population who are ethnic minorities, DEG is the percent of population with qualification level 4 or above, INC is the average gross disposable household income, OCC is the percent of the working population who are in managerial or professional occupations, a_i is the unobserved time-invariant individual effect and u_{it} is the error term.

The second model is a first-differences model. The full specification of which is:

$$\begin{aligned} MORT_{it} - MORT_{it-1} &= \beta(OUT_{it} - OUT_{it-1}) + \beta(\mathrm{LASPEND}_{it} - \mathrm{LASPEND}_{it-1}) \\ &+ \beta(\mathrm{CCGSPEND}_{it} - \mathrm{CCGSPEND}_{it-1}) + \beta(\mathrm{CLAIM}_{it} - \mathrm{CLAIM}_{it-1}) \\ &+ \beta(\mathrm{POP}_{it} - \mathrm{POP}_{it-1}) + \beta(\mathrm{UNEMP}_{it} - \mathrm{UNEMP}_{it-1}) \\ &+ \beta(\mathrm{ETHMIN}_{it} - \mathrm{ETHMIN}_{it-1}) + \beta(\mathrm{DEG}_{it} - \mathrm{DEG}_{it-1}) + \beta(\mathrm{INC}_{it} - \mathrm{INC}_{it-1}) \\ &+ \beta(\mathrm{OCC}_{it} - \mathrm{OCC}_{it-1}) + (\alpha_i - \alpha_i) + (\mu_{it} - \mu_{it-1}) \end{aligned}$$

The third and fourth model are fixed effects models, where the observations are weighted based on the number of GPs in each CCG in model 3 and the treatable mortality rate in 2013 for model 4. We use the non-parametric covariate balancing using propensity scores method advocated by Fong, Hazlett and Imai (2018). In which the weights, $w_{i,}$ for observations are (stabilized) inverse generalized propensity score weights. They are specified as

$$w_i = \frac{\mathrm{f}\,(\mathrm{T}_i^*)}{\mathrm{f}\,(\mathrm{T}_i^* \,|\mathrm{X}_i^*)'}$$

In which T is our treatment of for-profit outsourcing and X is our covariates of number of GPs and treatable mortality rate in 2013. $\mathrm{f}\,(\mathrm{T}_i^*)$ represents the marginal distribution of treatments and $\mathrm{f}\,(\mathrm{T}_i^* \,|\mathrm{X}_i^*)'$ represents the generalised propensity score.

The final model, model 5, is a random intercepts multilevel model where the individual, i, is Local authority, within a group of CCGs denoted as j at time t.

$$MORT_{ijt} = \beta OUT_{jt} + \beta LASPEND_{ijt} + \beta CCGSPEND_{jt} + \beta CLAIM_{ijt} + \beta POP_{ijt} + \beta UNEMP_{ijt} + \beta ETHMIN_{ijt} + \beta DEG_{ijt} + \beta INC_{ijt} + \beta OCC_{ijt} + \alpha_{jt} + \mu_{ijt}$$

Where α_{jt} represents the CCG random effect residual and μ_{ijt} the individual LA effect residual.

S.3. Models without log transformations of Treatable Mortality

To calculate an absolute change to mortality rate associated with changes in outsourcing, we run our models without any log transformation to the treatable mortality rate. We present the results below

	T. Mortality				
	FE	FD	CBPS (1)	CBPS (2)	MLM
For-profit Outsourcing (%)	0.291**	0.340**	0.281*	0.297**	0.193*
	(0.074)	(0.066)	(0.082)	(0.082)	(0.095)
	[0.094, 0.488]	[0.092, 0.587]	[0.067, 0.495]	[0.087, 0.508]	[0.007, 0.379]
LA Spend (£000s per person)	0.891	-0.066	1.222	0.879	1.847
	(1.872)	(2.258)	(6.223)	(6.085)	(1.318)
	[-4.107, 5.889]	[-5.258, 5.125]	[-7.678, 10.122]	[-7.850, 9.608]	[-0.737, 4.430]
Total CCG Spend (£Ms)	0.030	0.068	0.017	0.003	-0.007
	(0.057)	(0.072)	(0.058)	(0.054)	(0.009)
	[-0.066, 0.126]	[-0.045, 0.182]	[-0.097, 0.131]	[-0.103, 0.108]	[-0.025, 0.011]
Claimant Rate (%)	0.460	-0.183	0.290	0.130	8.347***
	(1.867)	(1.817)	(1.953)	(1.904)	(0.786)
	[-2.085, 3.004]	[-2.389, 2.023]	[-2.476, 3.056]	[-2.546, 2.807]	[6.807, 9.888]
Population size	33.259	53.357	40.020	52.708	0.643
	(53.052)	(72.599)	(54.743)	(52.729)	(1.367)
	[-63.164, 129.681]	[-112.665, 219.379]	[-64.867, 144.908]	[-48.407, 153.823]	[-2.036, 3.323]
Unemployment Rate (%)	0.325	0.426	0.332	0.338	-0.111
	(0.282)	(0.331)	(0.304)	(0.297)	(0.238)
	[-0.207, 0.856]	[-0.108, 0.960]	[-0.236, 0.900]	[-0.215, 0.891]	[-0.577, 0.355]
Ethnic Minority (%)	0.100	0.238	0.036	0.062	0.675***
	(0.185)	(0.168)	(0.184)	(0.179)	(0.065)
	[-0.283, 0.484]	[-0.152, 0.627]	[-0.380, 0.452]	[-0.341, 0.465]	[0.548, 0.803]
Degree Education (%)	-0.034	-0.101	0.003	0.000	-0.276***
	(0.137)	(0.143)	(0.142)	(0.128)	(0.072)
	[-0.303, 0.235]	[-0.399, 0.196]	[-0.298, 0.305]	[-0.282, 0.281]	[-0.417, -0.134]
Average Disposable H.hold Income	-15.709	30.010	-17.041	-12.325	-25.750***
	(20.903)	(23.372)	(25.146)	(24.667)	(3.489)
	[-64.997, 33.579]	[-9.296, 69.316]	[-76.087, 42.005]	[-69.496, 44.845]	[-32.588, -18.911]
Managerial/Professional occupation (%)	-0.163	-0.115	-0.111	-0.116	-0.040
	(0.136)	(0.141)	(0.133)	(0.126)	(0.088)
	[-0.460, 0.134]	[-0.423, 0.192]	[-0.448, 0.225]	[-0.435, 0.203]	[-0.212, 0.131]
SD (Observations)					2.486
Num.Obs.	609	450	517	553	534
R2	0.029	0.037	0.890	0.889	
R2 Adj.	-0.357	0.015	0.847	0.845	
R2 Marg.					0.715
R2 Cond.					0.815
AIC			3541.4	3774.6	3566.6
BIC			4170.2	4452.2	3635.1
ICC					0.4
Log.Lik.			-1622.724	-1730.320	
RMSE					5.91
CCG Fixed Effects	Yes	Yes	Yes	Yes	No
Time Fixed Effects	Yes	Yes	Yes	Yes	Yes
Clustered Standard Errors	Yes	Yes	Yes	Yes	Yes

+ $p < 0.1$, * $p < 0.05$, ** $p < 0.01$, *** $p < 0.001$

Outsourcing, LA Spend, and CCG Spend have a one year lag.

Population and GDHI are log transformed.

For full model expressions see supplementary material (sX)

Robust SEs are clustered at individual level and use a bias-reduced linearization estimator (CR2)

Satterthwaite degrees of freedom used in MLM

S.4. Standard Industrial Class

S.4.1 Division Groupings

We check whether this association is driven by outsourcing to companies with specific Standard Industrial Classification divisions. We aggregate divisions which may form a similar type of business. We find that outsourcing to companies classified as 'Human health activities' are statistically significantly associated with increases in treatable mortality rates.

In the below table: 'Human Health' refers to SIC division 86; 'Professional Services' refers to SIC divisions 70, 82, 62, 69, 74, 63 and 86; 'Building construction and maintenance' refers to SIC codes 68 and 41; 'Social Care' refers to SIC divisions 87 and 88; 'Foundational services' refers to SIC codes 49 and 56; and 'Other' refers to all other SIC codes combined. Control variables are just excluded from the table, included in the models.

	ln(T. Mortality)					
	**(1)**	**(2)**	**(3)**	**(4)**	**(5)**	**(6)**
For-profit Human Health Outsourcing (%)	0.005**					
	(0.001)					
	[0.001, 0.008]					
For-profit Professional services Outsourcing (%)		0.003				
		(0.002)				
		[-0.002, 0.008]				
For-profit 'Other' Outsourcing (%)			0.002			
			(0.002)			
			[-0.005, 0.009]			
For-profit Building construction and maintenance Outsourcing (%)				0.006+		
				(0.003)		
				[0.000, 0.012]		
For-profit Social Care Outsourcing (%)					-0.001	
					(0.012)	
					[-0.020, 0.018]	
For-profit Foundational Services Outsourcing (%)						0.006
						(0.002)
						[-0.006, 0.017]
Num.Obs.	609	609	609	609	609	609
R2	0.031	0.017	0.015	0.021	0.014	0.016
R2 Adj.	-0.354	-0.374	-0.377	-0.368	-0.378	-0.375
CCG Fixed Effects	Yes	Yes	Yes	Yes	Yes	Yes
Time Fixed Effects	Yes	Yes	Yes	Yes	Yes	Yes
Clustered Standard Errors	Yes	Yes	Yes	Yes	Yes	Yes
Control Variables	Yes	Yes	Yes	Yes	Yes	Yes

+ p < 0.1, * p < 0.05, ** p < 0.01, *** p < 0.001

Outsourcing, LA Spend, and CCG Spend have a one year lag.

Tr. mortaility, Population and GDHI are log transformed, 'Ln' denotes the natural log of outcome variable.

Robust SEs are clustered at individual level and use a bias-reduced linearization estimator (CR2)

S.4.2 Classes

We also analyse the lowest level of SIC within the 'Human Health' division to see if there are very specific types of healthcare business whose services are associated with mortality rates. At this scale the distinction between some of the classes is not entirely obvious and many businesses have multiple SIC codes which will include several within the human health division.

We find that "General medical practice activities" have a statistically significant positive association with treatable mortality rates:

	ln(T. Mortality)					
	**(1)**	**(2)**	**(3)**	**(4)**	**(5)**	**(6)**
For-profit Hospital Outsourcing (%)	0.006					
	(0.003)					
	[-0.003, 0.015]					
For-profit Dental Outsourcing (%)		-0.110				
		(0.265)				
		[-0.486, 0.266]				
For-profit Medical Nursing Homes Outsourcing (%)			-0.040			
			(0.018)			
			[-0.100, 0.019]			
For-profit Specialist Services Outsourcing (%)				-0.032		
				(0.020)		
				[-0.079, 0.016]		
For-profit General Medical Outsourcing (%)					0.005*	
					(0.002)	
					[0.001, 0.009]	
For-profit Other health Outsourcing (%)						0.003
						(0.003)
						[-0.003, 0.010]
Num.Obs.	609	609	609	609	609	609
R2	0.018	0.015	0.018	0.018	0.026	0.016
R2 Adj.	-0.372	-0.377	-0.373	-0.373	-0.362	-0.375
CCG Fixed Effects	Yes	Yes	Yes	Yes	Yes	Yes
Time Fixed Effects	Yes	Yes	Yes	Yes	Yes	Yes
Clustered Standard Errors	Yes	Yes	Yes	Yes	Yes	Yes
Control Variables	Yes	Yes	Yes	Yes	Yes	Yes

+ p < 0.1, * p < 0.05, ** p < 0.01, *** p < 0.001

Outsourcing, LA Spend, and CCG Spend have a one year lag.

Tr. mortaility, Population and GDHI are log transformed, 'Ln' denotes the natural log of outcome variable.

Robust SEs are clustered at individual level and use a bias-reduced linearization estimator (CR2)

Satterthwaite degrees of freedom used in MLM

S.4.3 Removing General Medical Practices

To be sure that our entire finding is not driven by a spurious relationship between General Medical Practices and Treatable Mortality, we remove these observations from the overall outsourcing data and rerun the main regression. We find consistent results and can conclude that the relationship is not entirely explained by expenditure on general medical practices.

	**ln(T. Mortality)**
	**(1)**
For-profit Outsourcing (General Medical Practices removed) (%)	0.00308***
	(0.00057)
	[0.00131, 0.00484]
Num.Obs.	609
R2	0.040
R2 Adj.	-0.342
CCG Fixed Effects	Yes
Time Fixed Effects	Yes
Clustered Standard Errors	Yes
Control Variables	Yes

+ p < 0.1, * p < 0.05, ** p < 0.01, *** p < 0.001

Outsourcing, LA Spend, and CCG Spend have a one year lag.

Tr. mortaility, Population and GDHI are log transformed, ‘Ln’ denotes the natural log of outcome variable.

Robust SEs are clustered at individual level and use a bias-reduced linearization estimator (CR2)

S.5 Time Fixed Effects

Our main results include fixed effects for year and CCG. Here we present the coefficients of the fixed effects for year and the intercept in the full model. '2014' will be treated as reference categories – bearing in mind we regress 2013 outsourcing against 2014 mortality rates, so we do not have a 2013 year.

	**ln(T. Mortality)**
	**FE**
(Intercept)	0.60057
	(7.69460)
	[-13.10224, 14.30338]
	[-0.00554, 0.00105]
factor(year)2015	0.00871
	(0.02100)
	[-0.03026, 0.04767]
factor(year)2016	0.00814
	(0.02489)
	[-0.03855, 0.05482]
factor(year)2017	-0.01279
	(0.03109)
	[-0.06981, 0.04422]
factor(year)2018	-0.00775
	(0.04286)
	[-0.08641, 0.07091]
factor(year)2019	-0.05880
	(0.05233)
	[-0.15362, 0.03602]
Num.Obs.	609
R2	0.890
R2 Adj.	0.846
AIC	-1322.7
BIC	-550.7
Log.Lik.	836.361
CCG Fixed Effects	Yes
Time Fixed Effects	Yes
Clustered Standard Errors	Yes

+ p < 0.1, * p < 0.05, ** p < 0.01, *** p < 0.001

Outsourcing, LA Spend, and CCG Spend have a one year lag.

Tr. Mortality, Population and GDHI are log transformed, 'Ln' denotes the natural log of outcome variable.

For full model expressions see supplementary material (sX)

Robust SEs are clustered at individual level and use a bias-reduced linearization estimator (CR2)

S.6. Nonparametric Covariate Balancing using Propensity Scores

In our main results we present two models (models 3 and 4) which include covariate balancing using propensity scores. The idea of these models is to balance the sample based on key variables – as would be done were analysis to be done on a treatment and control group – but with a continuous 'treatment' of for-profit outsourcing.

We conduct analyses using the CBPS package in R. Covariate balancing is an advanced matching method which can weight values to balance the model, accounting for differences in observations according to their value of a continuous treatment variable, in this case for-profit outsourcing.

We balance our sample firstly on the number of doctors in any given CCG and secondly on the treatable mortality rate at the beginning of our time series in 2013. Below we present the balance plots of how the weights are applied by the propensity scores. 'Treat' refers to the 'treatment' of for-profit outsourcing (%).

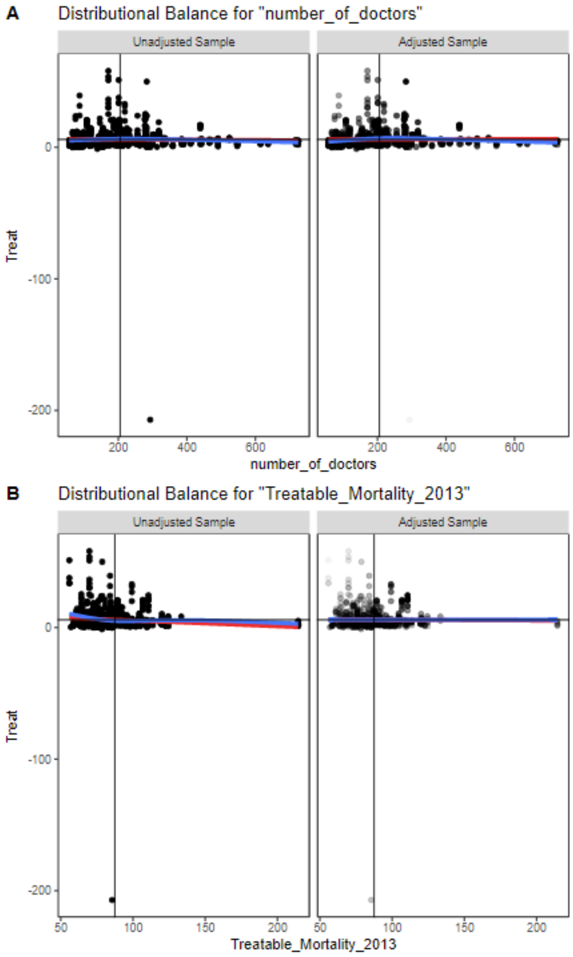

S.7. Regression on absolute numbers of deaths

We are interested in finding out how many additional deaths can be attributed to changes in outsourcing. To do this we need to run models on the number of treatable deaths - as opposed to the age-standardised rate per 100,000 population. In these models we control for age (% aged over 70) as the dependent variable is no longer age-standardised – and treatable deaths are only counted for people aged 0-74.

For calculating our additional deaths, we need to work out annual additional deaths attributable to for-profit outsourcing. When creating about cumulative changes, calculating additional total spend is more meaningful than the cumulative additional % of spend so we run a model with total spend on absolute deaths.

In the table below model 1 shows our full model regression for-profit outsourcing against number of deaths. Model 2 presents the same model but changing the independent variable of interest to absolute expenditure on for profit companies. Model 2 will have some additional variation in the expenditure variable because we have two partial years of data. This means the change from 2013 to 2014 is not accurate. Consequently, in model 3 we drop our expenditure data years of 2013 and 2020 for which we have partial observations. The coefficient and confidence intervals in model 3 is what we use to create figure 2 in the paper.

		**Treatable Deaths (n)**	
	**(1)**	**(2)**	**(3)**
For-profit Outsourcing (%)	0.5689**		
	(0.1375)		
	[0.1486, 0.9892]		
Private sector spend (£ms)		0.2886*	0.2910*
		(0.1002)	(0.1066)
		[0.0635, 0.5138]	[0.0513, 0.5306]
Num.Obs.	609	609	519
R2	0.049	0.047	0.032
R2 Adj.	-0.332	-0.335	-0.441
CCG Fixed Effects	Yes	Yes	Yes
Time Fixed Effects	Yes	Yes	Yes
Clustered Standard Errors	Yes	Yes	Yes
Control variables	Yes	Yes	Yes

+ p < 0.1, * p < 0.05, ** p < 0.01, *** p < 0.001

Outsourcing/Spend, LA Spend, and CCG Spend have a one year lag.

Population and GDHI are log transformed

Robust SEs are clustered at individual level and use a bias-reduced linearization estimator (CR2)

S.8 Additional deaths plot

In the paper we present figure 2 which is plots the additional deaths attributed to for-profit outsourcing. In the main paper we crop this to 2018 to keep as many CCGs included as possible. We do have data to extent this to 2019 but we lose observations because the mortality data is only available for CCGs which existed after the significant mergers in April 2020. We cannot extend it to 2020 because we only have partial expenditure data for 2020, so additional rates of for-profit expenditure are all negative superficially. Below we present the figure up to 2019 with the 51 CCGs which we have consistent data for. We see that treatable mortality declined severely in 2019. 2020 data appears to show a rise back to 2018 levels so this may not be a sustained improvement (COVID-19 itself is not considered a 'treatable' cause of death but undoubtedly had a huge effect on health service performance).

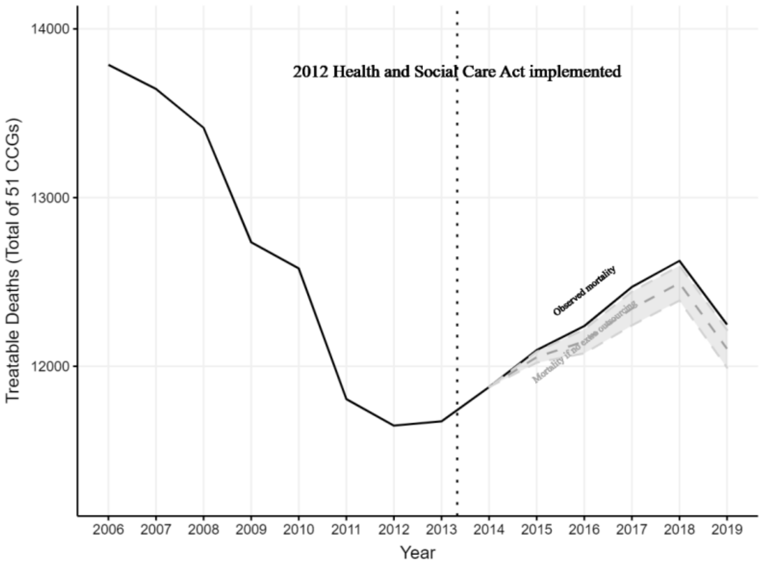

Sensitivity Checks

S.9. Falsification test

To ensure that our findings are not measuring some overall population health, rather than healthcare quality, we run our regressions with the outcome variable of preventable mortality – deaths from causes that are possible to be avoided through public health intervention. We find no statistically significant results and can be more confident our main finding represents changes to some form of healthcare quality rather than an association with societal breakdown.

	ln(P. Mortality)				
	**FE**	**FD**	**CBPS (1)**	**CBPS (2)**	**MLM**
For-profit Outsourcing (%)	0.0013	0.0021+	0.0009	0.0009	0.0017
	(0.0008)	(0.0015)	(0.0008)	(0.0008)	(0.0013)
	[-0.0006, 0.0031]	[-0.0002, 0.0045]	[-0.0010, 0.0028]	[-0.0011, 0.0028]	[-0.0009, 0.0043]
LA Spend (£000s per person)	0.0214	0.0344	-0.0222	-0.0127	0.0340+
	(0.0282)	(0.0151)	(0.0369)	(0.0380)	(0.0184)
	[-0.0248, 0.0676]	[-0.0149, 0.0837]	[-0.1024, 0.0579]	[-0.0928, 0.0675]	[-0.0020, 0.0700]
Total CCG Spend (£Ms)	0.0006	0.0013*	-0.0002	0.0001	-0.0001
	(0.0004)	(0.0004)	(0.0004)	(0.0004)	(0.0001)
	[-0.0003, 0.0015]	[0.0002, 0.0024]	[-0.0013, 0.0008]	[-0.0009, 0.0011]	[-0.0003, 0.0002]
Claimant Rate (%)	0.0057	0.0084	0.0076	0.0042	0.1309***
	(0.0093)	(0.0117)	(0.0107)	(0.0104)	(0.0109)
	[-0.0178, 0.0292]	[-0.0126, 0.0294]	[-0.0173, 0.0325]	[-0.0204, 0.0288]	[0.1095, 0.1523]
Population size	-0.0097	0.0970	0.1212	-0.0110	0.0000
	(0.5388)	(0.6812)	(0.5279)	(0.5179)	(0.0190)
	[-0.9010, 0.8817]	[-1.4811, 1.6750]	[-0.8237, 1.0660]	[-0.9393, 0.9172]	[-0.0372, 0.0373]
Unemployment Rate (%)	-0.0009	-0.0004	-0.0013	-0.0015	0.0007
	(0.0028)	(0.0026)	(0.0027)	(0.0027)	(0.0033)
	[-0.0058, 0.0040]	[-0.0054, 0.0047]	[-0.0065, 0.0038]	[-0.0066, 0.0036]	[-0.0058, 0.0071]
Ethnic Minority (%)	-0.0017	-0.0026	-0.0018	-0.0021	0.0040***
	(0.0018)	(0.0022)	(0.0020)	(0.0019)	(0.0009)
	[-0.0052, 0.0019]	[-0.0063, 0.0011]	[-0.0056, 0.0019]	[-0.0058, 0.0016]	[0.0022, 0.0057]
Degree Education (%)	-0.0002	-0.0008	0.0002	0.0005	0.0004
	(0.0013)	(0.0014)	(0.0013)	(0.0012)	(0.0010)
	[-0.0027, 0.0023]	[-0.0037, 0.0020]	[-0.0025, 0.0030]	[-0.0021, 0.0031]	[-0.0016, 0.0023]
Average Disposable H.hold Income	-0.1101	-0.0294	0.2336	0.0918	-0.5838***
	(0.2725)	(0.2379)	(0.3660)	(0.3561)	(0.0486)
	[-0.5657, 0.3456]	[-0.4031, 0.3442]	[-0.2983, 0.7655]	[-0.4330, 0.6167]	[-0.6791, -0.4885]
Managerial/Professional occupation (%)	-0.0007	-0.0022	0.0010	0.0004	-0.0026*
	(0.0020)	(0.0015)	(0.0018)	(0.0018)	(0.0012)
	[-0.0034, 0.0021]	[-0.0051, 0.0007]	[-0.0020, 0.0041]	[-0.0025, 0.0034]	[-0.0049, -0.0002]
SD (Observations)					0.2924
Num.Obs.	609	450	517	553	534
R2	0.014	0.037	0.953	0.951	
R2 Adj.	-0.378	0.015	0.935	0.932	
R2 Marg.					0.766
R2 Cond.					0.853
AIC			-1328.3	-1413.3	-881.7
BIC			-699.6	-735.8	-813.2
ICC					0.4
Log.Lik.			812.151	863.633	
RMSE					0.08
CCG Fixed Effects	Yes	Yes	Yes	Yes	No
Time Fixed Effects	Yes	Yes	Yes	Yes	Yes
Clustered Standard Errors	Yes	Yes	Yes	Yes	Yes

+ p < 0.1, * p < 0.05, ** p < 0.01, *** p < 0.001

Outsourcing, LA Spend, and CCG Spend have a one year lag.

P. Mortality, Population and GDHI are log transformed, 'Ln' denotes the natural log of outcome variable.

Robust SEs are clustered at individual level and use a bias-reduced linearization estimator (CR2)

Satterthwaite degrees of freedom used in MLM

S.10. Dropping individual CCGs

S.10.1 Plotting results

To ensure that our overall finding isn't driven by a single CCG we sequentially drop each CCG from our model. We find that removing any single CCG only changes the coefficient minimally and the results are always statistically significant.

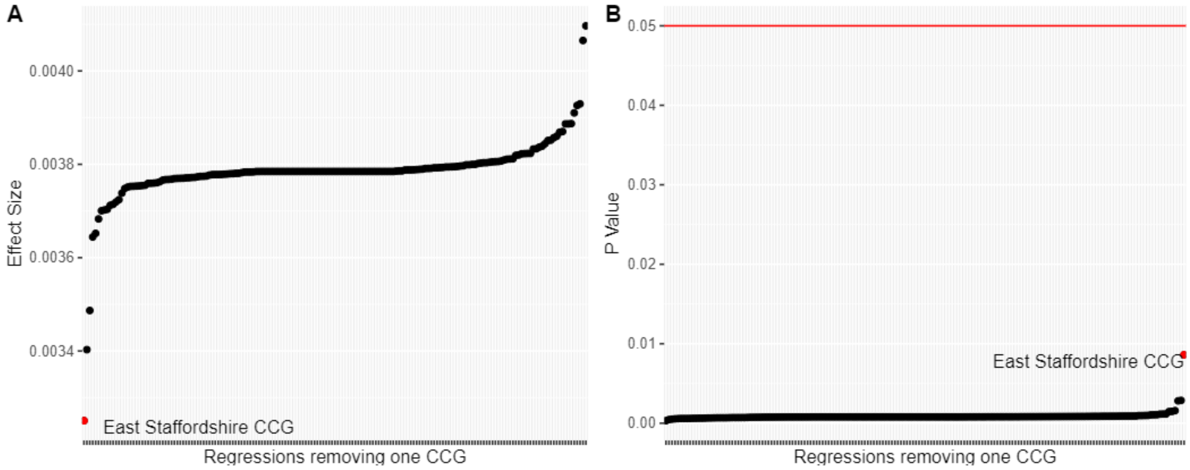

S.10.2. Full Model removing East Staffordshire

To ensure that our model is completely robust, we removed East Staffordshire giving us the lowest possible coefficient and highest possible p-value, then ran our models with robust standard errors to make entirely sure that our findings hold given any missing CCG.

	**ln(Tr. Mortality)**			
	**FE**	**FD**	**CBPS (1)**	**CBPS (2)**
For-profit Outsourcing (%)	0.0033**	0.0045**	0.0043*	0.0034**
	(0.0008)	(0.0014)	(0.0018)	(0.0009)
	[0.0009, 0.0057]	[0.0016, 0.0074]	[0.0001, 0.0086]	[0.0008, 0.0059]
LA Spend (£000s per person)	0.0028	-0.0023	0.0002	-0.0064
	(0.0203)	(0.0191)	(0.0725)	(0.0617)
	[-0.0526, 0.0582]	[-0.0601, 0.0555]	[-0.1037, 0.1042]	[-0.1010, 0.0883]
Total CCG Spend (£Ms)	0.0004	0.0008	0.0013	0.0000
	(0.0007)	(0.0008)	(0.0008)	(0.0006)
	[-0.0007, 0.0014]	[-0.0005, 0.0020]	[-0.0003, 0.0028]	[-0.0012, 0.0011]
Claimant Rate (%)	0.0133	0.0009	0.0310	0.0098
	(0.0165)	(0.0149)	(0.0204)	(0.0171)
	[-0.0149, 0.0415]	[-0.0237, 0.0254]	[-0.0095, 0.0715]	[-0.0192, 0.0389]
Population size	0.4770	0.8071	0.2513	0.7247
	(0.6720)	(0.8091)	(0.8628)	(0.5539)
	[-0.5927, 1.5467]	[-1.0417, 2.6560]	[-1.1763, 1.6790]	[-0.3728, 1.8222]
Unemployment Rate (%)	0.0023	0.0044	0.0050	0.0023
	(0.0030)	(0.0041)	(0.0043)	(0.0032)
	[-0.0036, 0.0082]	[-0.0015, 0.0104]	[-0.0029, 0.0129]	[-0.0038, 0.0083]
Ethnic Minority (%)	0.0023	0.0039+	0.0036	0.0016
	(0.0024)	(0.0017)	(0.0027)	(0.0021)
	[-0.0020, 0.0066]	[-0.0005, 0.0083]	[-0.0022, 0.0094]	[-0.0028, 0.0060]
Degree Education (%)	-0.0008	-0.0015	-0.0017	-0.0003
	(0.0015)	(0.0012)	(0.0022)	(0.0015)
	[-0.0038, 0.0022]	[-0.0048, 0.0019]	[-0.0063, 0.0029]	[-0.0034, 0.0028]
Average Disposable H.hold Income	-0.1619	0.3290	-0.4489	-0.1097
	(0.2640)	(0.1704)	(0.4015)	(0.2785)
	[-0.7084, 0.3847]	[-0.1104, 0.7684]	[-1.2787, 0.3810]	[-0.7299, 0.5105]
Managerial/Professional occupation (%)	-0.0022	-0.0018	-0.0016	-0.0013
	(0.0018)	(0.0016)	(0.0024)	(0.0015)
	[-0.0055, 0.0011]	[-0.0052, 0.0017]	[-0.0061, 0.0030]	[-0.0047, 0.0022]
Num.Obs.	604	446	370	548
R2	0.032	0.047	0.899	0.894
R2 Adj.	-0.354	0.025	0.843	0.852
AIC			-810.1	-1217.6
BIC			-289.6	-545.8
Log.Lik.			538.033	764.803
CCG Fixed Effects	Yes	Yes	Yes	Yes
Time Fixed Effects	Yes	Yes	Yes	Yes
Clustered Standard Errors	Yes	Yes	Yes	Yes

+ p < 0.1, * p < 0.05, ** p < 0.01, *** p < 0.001

Outsourcing, LA Spend, and CCG Spend have a one year lag.

Tr. Mortality, Population and GDHI are log transformed, 'Ln' denotes the natural log of outcome variable.

Robust SEs are clustered at individual level and use a bias-reduced linearization estimator (CR2)

S.11. Specification curve

To ensure that our finding is not driven by the specific choice of covariates we run a specification curve and find that the effect size varies only minimally with any combination of our covariates. All results have a significant P value, smaller than 0.05. The effect-size varies from 0.00387 to 0.00404 in any given specification which is very stable and suggests our findings are by no means determined by the selection of covariates.

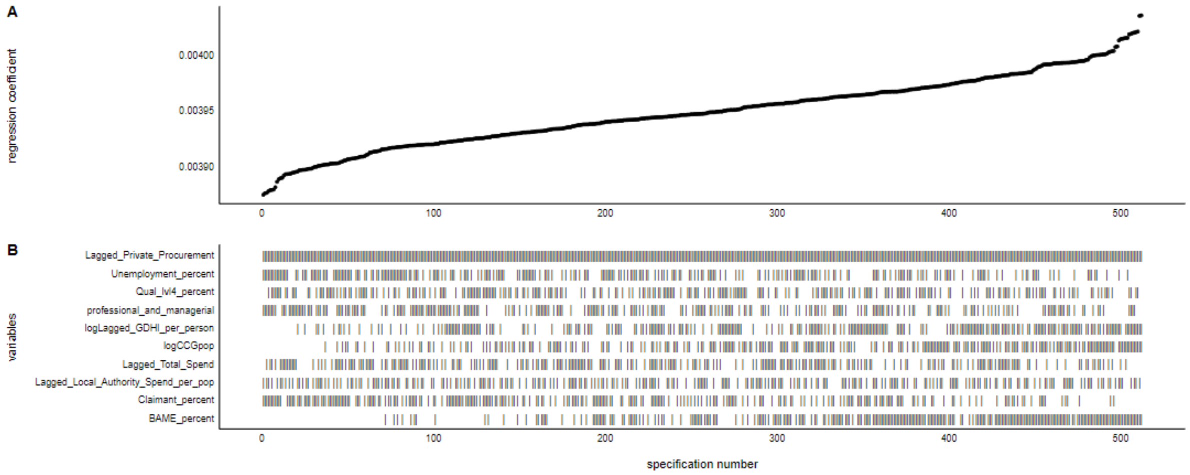

S.12. Combined Specification curve and random error simulation

S.12.1. Plotting all specification coefficients together

We combine the specification curve with our random error simulation – looping each specification with varying levels of random error. Here we ran each of the 512 specifications 100 times with 5 different error sizes (20 times with each error size). Below we plot the coefficients and significance levels for each of these regressions.

This analysis shows sensitivity in that it represents how much random error would be needed in our data for our results not to hold – or – how much random error could be in our data in which it still holds. The model used is the same two way FE model which does not account for spatial effects.

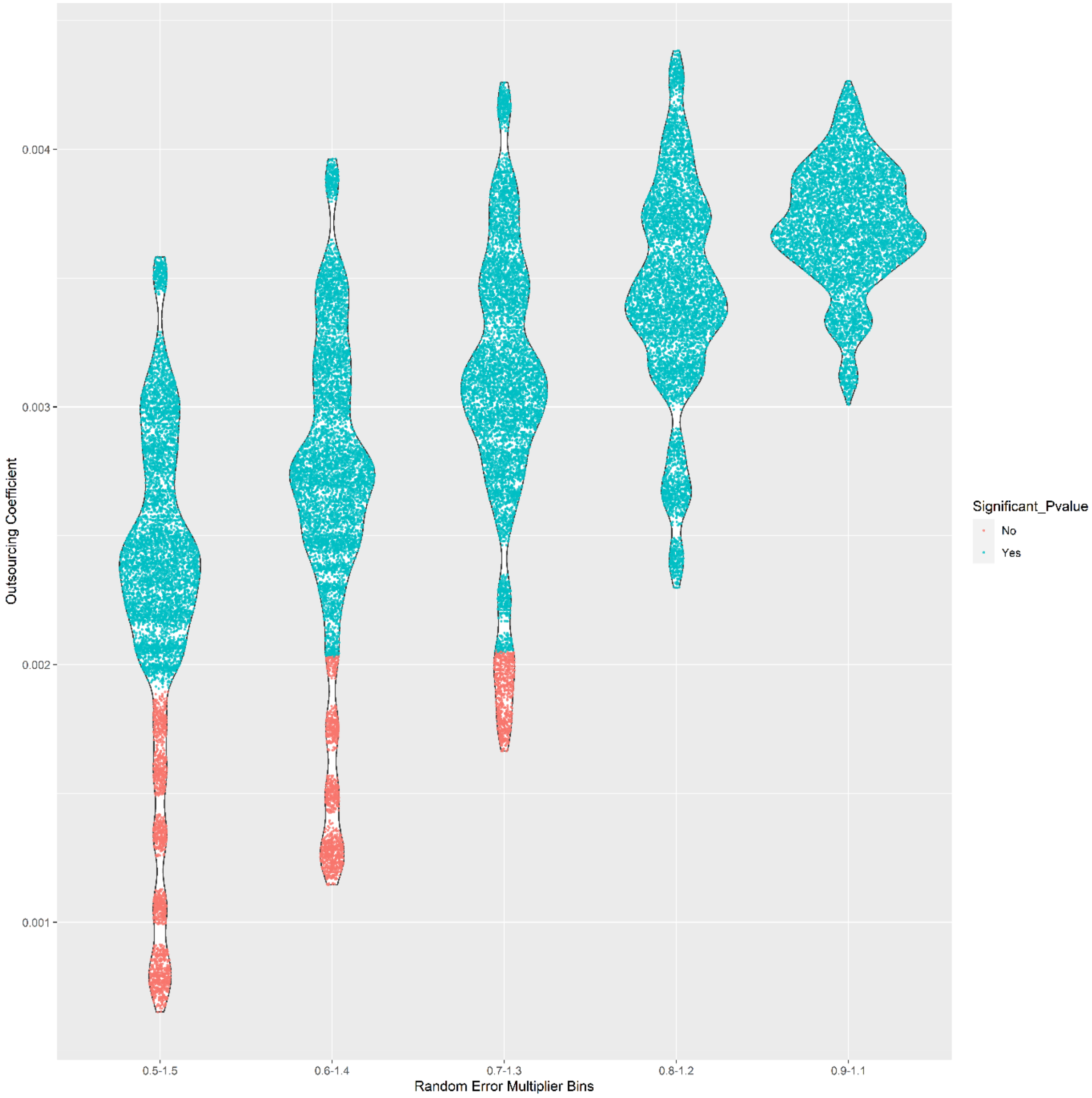

S.12.2. Selected specifications

If we separate the random error loops for each specification, we can see that given any single specification, the random error creates very similar results. Below we select 17 representatively from the whole sample of specifications, based on average coefficient sizes.

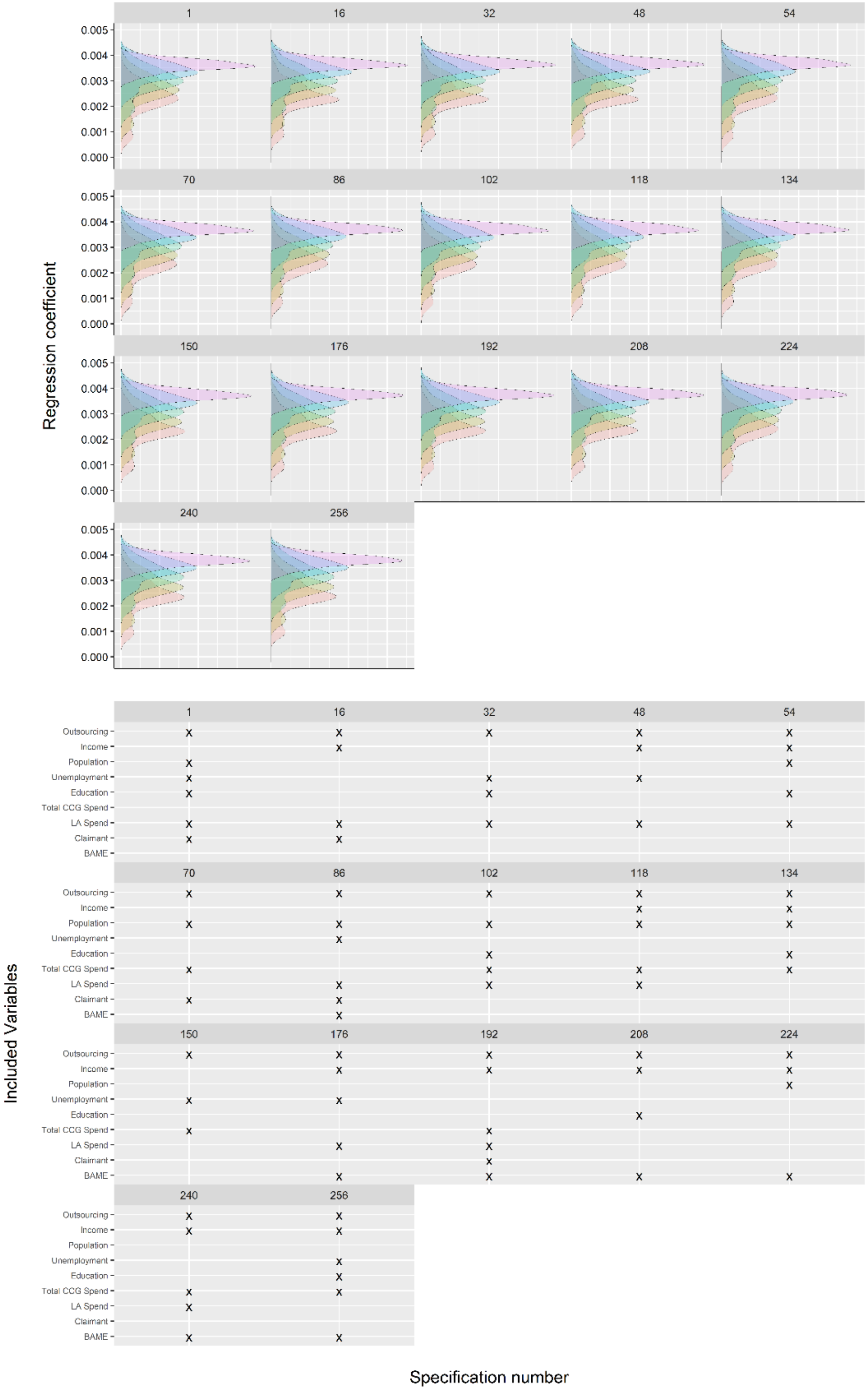

S.13. Matching

In our main results we report outcomes from covariate balancing using nonparametric methods with propensity scores. This is the best method for our data because we have a continuous 'treatment' variable – outsourcing %. We can also wrangle outsourcing to be a binary treatment – CCGs either experience a high level of outsourcing or a low level of outsourcing – ie. they are grouped into a treatment group if they experience some level of outsourcing, or a control group if they do not. In this way we can weight observations based on full matches on the number of GPs and Treatable Mortality Rates in 2013.

Using this identification, we can run more traditional, full matching models. The models have a very similar effect size to our npCBPS. In the table below, models 1-3 are matching on the number of GPs and models 4-6 are matching on levels of Treatable Mortality in 2013. Models 1 and 4 treat 4% of for-profit outsourcing as the treatment benchmark, models 2 and 5 use 7% and models 3 and 6 use 10%.

	**ln(T. Mortality)**					
	**(1)**	**(2)**	**(3)**	**(4)**	**(5)**	**(6)**
For-profit Outsourcing (%)	0.0037***	0.0035***	0.0038***	0.0033**	0.0040***	0.0035**
	(0.0008)	(0.0008)	(0.0009)	(0.0010)	(0.0010)	(0.0010)
	[0.0017, 0.0057]	[0.0016, 0.0054]	[0.0018, 0.0057]	[0.0010, 0.0056]	[0.0022, 0.0059]	[0.0013, 0.0056]
Num.Obs.	571	571	571	571	571	571
R2	0.912	0.930	0.913	0.893	0.926	0.910
R2 Adj.	0.877	0.903	0.879	0.851	0.897	0.874
AIC	-1213.1	-1097.6	-1196.5	-839.0	-974.9	-839.6
BIC	-500.2	-384.7	-483.5	-126.0	-261.9	-126.6
Log.Lik.	770.575	712.813	762.225	583.496	651.438	583.786
CCG Fixed Effects	Yes	Yes	Yes	Yes	Yes	Yes
Time Fixed Effects	Yes	Yes	Yes	Yes	Yes	Yes
Clustered Standard Errors	Yes	Yes	Yes	Yes	Yes	Yes
Control Variables	Yes	Yes	Yes	Yes	Yes	Yes

+ p < 0.1, * p < 0.05, ** p < 0.01, *** p < 0.001

Outsourcing, LA Spend, and CCG Spend have a one year lag.

Tr. mortaility, Population and GDHI are log transformed, 'Ln' denotes the natural log of outcome variable.

Robust SEs are clustered at individual level and use a bias-reduced linearization estimator (CR2)

Additional Analyses

S.14. Quadratic term

To test whether our result has a non-linear relationship. We find no significant result with our polynomial variable.

	**ln(T. Mortality)**
	**(1)**
For-profit Outsourcing (%)	0.0029
	(0.0021)
	[-0.0012, 0.0071]
Quadratic Outsourcing Term	0.0000
	(0.0000)
	[-0.0001, 0.0001]
Num.Obs.	609
R2	0.890
R2 Adj.	0.846
AIC	-1321.1
BIC	-544.6
Log.Lik.	836.535
CCG Fixed Effects	Yes
Time Fixed Effects	Yes
Clustered Standard Errors	Yes
Control Variables	Yes

+ p < 0.1, * p < 0.05, ** p < 0.01, *** p < 0.001

Outsourcing, LA Spend, and CCG Spend have a one year lag.

Tr. mortaility, Population and GDHI are log transformed, 'Ln' denotes the natural log of outcome variable.

Robust SEs are clustered at individual level and use a bias-reduced linearization estimator (CR2)

S.14. Growth curve models

S.14.1 Unconditional growth model

We are interested to see if Outsourcing increased statistically significantly over this period. We find that, as per our descriptive analysis, for-profit outsourcing has indeed increased between 2013 and 2020.

	**For-profit Outsourcing (%)**
	**(1)**
Time	0.2321*
	(0.1166)
	[0.0034, 0.4607]
Num.Obs.	944
R2 Marg.	0.005
R2 Cond.	
AIC	6750.8
BIC	6779.9
RMSE	6.98
Clustered Standard Errors	Yes

+ p < 0.1, * p < 0.05, ** p < 0.01, *** p < 0.001

Tr. mortaility is log transformed, 'Ln' denotes the natural log of outcome variable.

Robust SEs are clustered at individual level and use a bias-reduced linearization estimator (CR2)

S.14.2 Conditional growth model

We are also interested if the absolute levels of outsourcing explain changes in mortality rates over this period. We find that levels of outsourcing do not explain changes in mortality.

	**ln(T. Mortality)**
	**(1)**
For-profit Outsourcing (%)	0.3984
	(0.4419)
	[-0.4678, 1.2645]
Time	-0.0055**
	(0.0019)
	[-0.0093, -0.0017]
Interaction term	-0.0002
	(0.0002)
	[-0.0006, 0.0002]
Num.Obs.	785
R2 Marg.	0.005
R2 Cond.	0.834
AIC	-1239.0
BIC	-1201.7
ICC	0.8
RMSE	0.07
Clustered Standard Errors	Yes
Control Variables	Yes

+ p < 0.1, * p < 0.05, ** p < 0.01, *** p < 0.001

Tr. mortaility is log transformed, 'Ln' denotes the natural log of outcome variable.

Robust SEs are clustered at individual level and use a bias-reduced linearization estimator (CR2)

S.15 Difference in Difference

Levels of outsourcing are not a binary treatment and almost all CCGs saw an increase in outsourcing between 2013 and 2020. There are only five CCGs which report no increases in outsourcing, but have at least four years of spending reported (three observations of spending change). As such, it is not possible to conduct a rigorous difference in differences analysis.

However below we run a DiD to see whether the coefficient is somewhat comparable to the main findings. We consider that CCGs which did not increase their outsourcing in any given year, but reported at least four years of data, are a control group and all other CCGs which report at least three years of data are a treatment group. We then look at treatable mortality rates before and after 2014 to compare whether the 'treatment' of increased outsourcing is associated with worse changes in mortality rates.

We find a positive statistically insignificant difference in difference between the CCGs which increased outsourcing in at least one year and those which had no increases in outsourcing at all.

S.15.1 DiD Table

	ln(T. Mortality)
	(1)
Treatment	0.1311**
	(0.0407)
	[0.0513, 0.2110]
Time	-0.0723
	(0.0530)
	[-0.1761, 0.0316]
Treatment*Time	0.0149
	(0.0541)
	[-0.0913, 0.1211]
Num.Obs.	1092
R2	0.048
R2 Adj.	0.045
AIC	-664.2
BIC	-639.2
F	18.311
RMSE	0.18

+ p < 0.1, * p < 0.05, ** p < 0.01, *** p < 0.001

S.15.2 DiD graph

We visualise this relationship below.

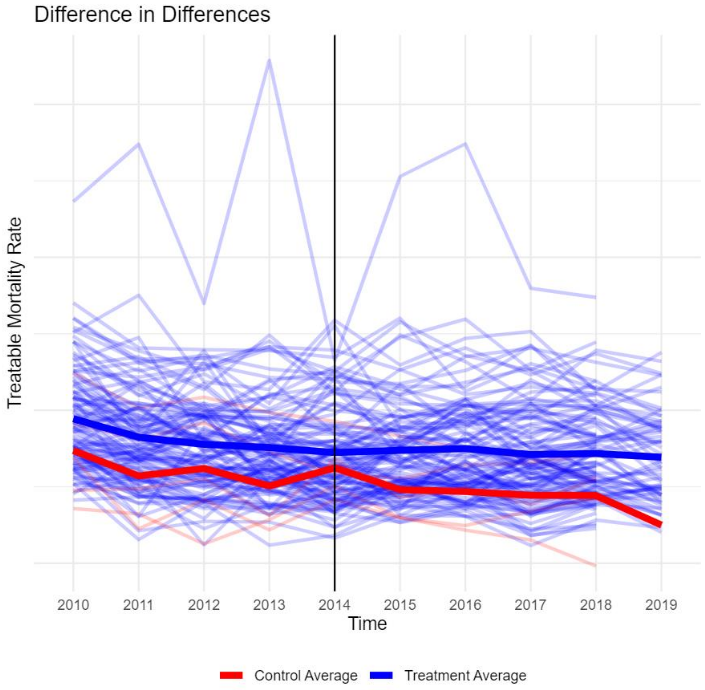

S.16. Interactions terms

We are interested in how the relationship between outsourcing and treatable mortality differs according to changes in austerity and population demographics. Perhaps the increase in mortality, given an increase in outsourcing is greater when the population is becoming poorer or total CCG expenditure is declining.

S.16.1 Interaction with austerity

We find no statistically significant interaction effect when testing whether changes to total CCG spend or LA spend alters the relationship between outsourcing and treatable mortality.

	**ln(T. Mortality)**	
	**(1)**	**(2)**
For-profit Outsourcing (%)	0.0033**	0.0035*
	(0.0007)	(0.0010)
	[0.0010, 0.0056]	[0.0007, 0.0063]
Total CCG Spend (£Ms)	0.0000	
	(0.0007)	
	[-0.0013, 0.0013]	
Outsourcing*CCG Spend	0.0000	
	(0.0001)	
	[-0.0001, 0.0002]	
LA spend (£000s per person)		0.0029
		(0.0250)
		[-0.0555, 0.0613]
Outsourcing*LA Spend		0.0000
		(0.0008)
		[-0.0021, 0.0022]
Num.Obs.	648	648
R2	0.886	0.886
R2 Adj.	0.846	0.846
AIC	-1429.3	-1428.8
BIC	-668.7	-668.3
RMSE	0.06	0.06
CCG Fixed Effects	Yes	Yes
Time Fixed Effects	Yes	Yes
Clustered Standard Errors	Yes	Yes

+ p < 0.1, * p < 0.05, ** p < 0.01, *** p < 0.001

Outsourcing, LA Spend, and CCG Spend have a one year lag.

Tr. mortaility, Population and GDHI are log transformed, 'Ln' denotes the natural log of outcome variable.

Robust SEs are clustered at individual level and use a bias-reduced linearization estimator (CR2)

S.16.2 Interaction with demographics

We also find no statistically significant interaction effect for any our demographic variables.

	ln(T. Mortality)						
	(1)	(2)	(3)	(4)	(5)	(6)	(7)
For-profit Outsourcing (%)	-0.0647	0.0168	-0.0021	0.0035*	0.0040*	0.0040+	-0.0019
	(0.0474)	(0.0260)	(0.0046)	(0.0011)	(0.0016)	(0.0018)	(0.0042)
	[-0.1533, 0.0239]	[-0.0502, 0.0838]	[-0.0124, 0.0082]	[0.0008, 0.0062]	[0.0003, 0.0078]	[-0.0003, 0.0082]	[-0.0114, 0.0075]
Average Disposable H.hold Income	-0.1505						
	(0.2416)						
	[-0.6624, 0.3614]						
Outsourcing*Income	0.0068						
	(0.0048)						
	[-0.0020, 0.0157]						
Population size		0.4897					
		(0.6013)					
		[-0.5815, 1.5610]					
Outsourcing*Population		-0.0011					
		(0.0021)					
		[-0.0065, 0.0044]					
Managerial/Professional occupation (%)			-0.0034*				
			(0.0020)				
			[-0.0068, 0.0000]				
Outsourcing*Occupation			0.0002				
			(0.0001)				
			[-0.0001, 0.0005]				
Ethnic Minority (%)				0.0019			
				(0.0021)			
				[-0.0025, 0.0062]			
Outsourcing*Ethnicity				0.0000			
				(0.0001)			
				[-0.0002, 0.0002]			
Claimant Rate (%)					0.0145		
					(0.0185)		
					[-0.0170, 0.0460]		
Outsourcing*Claimant Rate					-0.0002		
					(0.0010)		
					[-0.0026, 0.0021]		
Unemployment Rate (%)						0.0017	
						(0.0033)	
						[-0.0056, 0.0089]	
Outsourcing*Unemployment Rate						0.0000	
						(0.0003)	
						[-0.0009, 0.0008]	
Degree Education (%)							-0.0019
							(0.0017)
							[-0.0050, 0.0011]
Outsourcing*Education							0.0001
							(0.0001)
							[-0.0001, 0.0004]
Num.Obs.	609	609	609	609	609	609	609
R2	0.889	0.888	0.889	0.889	0.888	0.888	0.889
R2 Adj.	0.847	0.847	0.848	0.847	0.847	0.846	0.847
AIC	-1331.5	-1329.2	-1333.4	-1329.5	-1329.2	-1328.3	-1331.0
BIC	-590.3	-588.0	-592.2	-588.3	-588.0	-587.1	-589.8
RMSE	0.06	0.06	0.06	0.06	0.06	0.06	0.06
CCG Fixed Effects	Yes	Yes	Yes	Yes	Yes	Yes	Yes
Time Fixed Effects	Yes	Yes	Yes	Yes	Yes	Yes	Yes
Clustered Standard Errors	Yes	Yes	Yes	Yes	Yes	Yes	Yes

+ p < 0.1, * p < 0.05, ** p < 0.01, *** p < 0.001

Outsourcing, LA Spend, and CCG Spend have a one year lag.

Tr. mortaility, Population and GDHI are log transformed, ‘Ln’ denotes the natural log of outcome variable.

Robust SEs are clustered at individual level and use a bias-reduced linearization estimator (CR2)

S.16.3 Interaction between deprived and affluent CCGs

We can also conduct interaction effects with time invariant measure of deprivation in a mixed effects model to understand whether the relationship between outsourcing and treatable mortality differs according to overall level of deprivation. Here we use the Index of Multiple Deprivation 2019, extent of deprivation measure. Again we find no significant interaction.

	**ln(T. Mortality)**
	**(1)**
For-profit Outsourcing (%)	-0.0005
	(0.0010)
	[-0.0025, 0.0016]
Deprivation (2019)	0.8847***
	(0.0481)
	[0.7905, 0.9790]
Outsourcing*Deprivation	0.0040
	(0.0052)
	[-0.0062, 0.0142]
Num.Obs.	648
R2 Marg.	0.710
R2 Cond.	0.847
AIC	-1239.6
BIC	-1190.4
ICC	0.5
RMSE	0.07
CCG Random Effects	Yes
Time Fixed Effects	Yes
Clustered Standard Errors	Yes

+ p < 0.1, * p < 0.05, ** p < 0.01, *** p < 0.001

Outsourcing, LA Spend, and CCG Spend have a one year lag.

Tr. mortaility, Population and GDHI are log transformed, ‘Ln’ denotes the natural log of outcome variable.

Robust SEs are clustered at individual level and use a bias-reduced linearization estimator (CR2)

S.17. Spatial autocorrelation

Our results look at within CCG changes in outsourcing, however, it may be the case that there are clusters of high outsourcing. We assess whether, on overall % of outsourcing 2013-2020, there are clusters of high outsourcing. We present LISA clusters below analysing the spatial autocorrelation between bordering neighbours.

We find two clusters of high outsourcing levels, statistically significant at the 5% level. One region in West of England and Nottingham city. However overall, there is very little spatial autocorrelation in the levels of for-profit outsourcing.

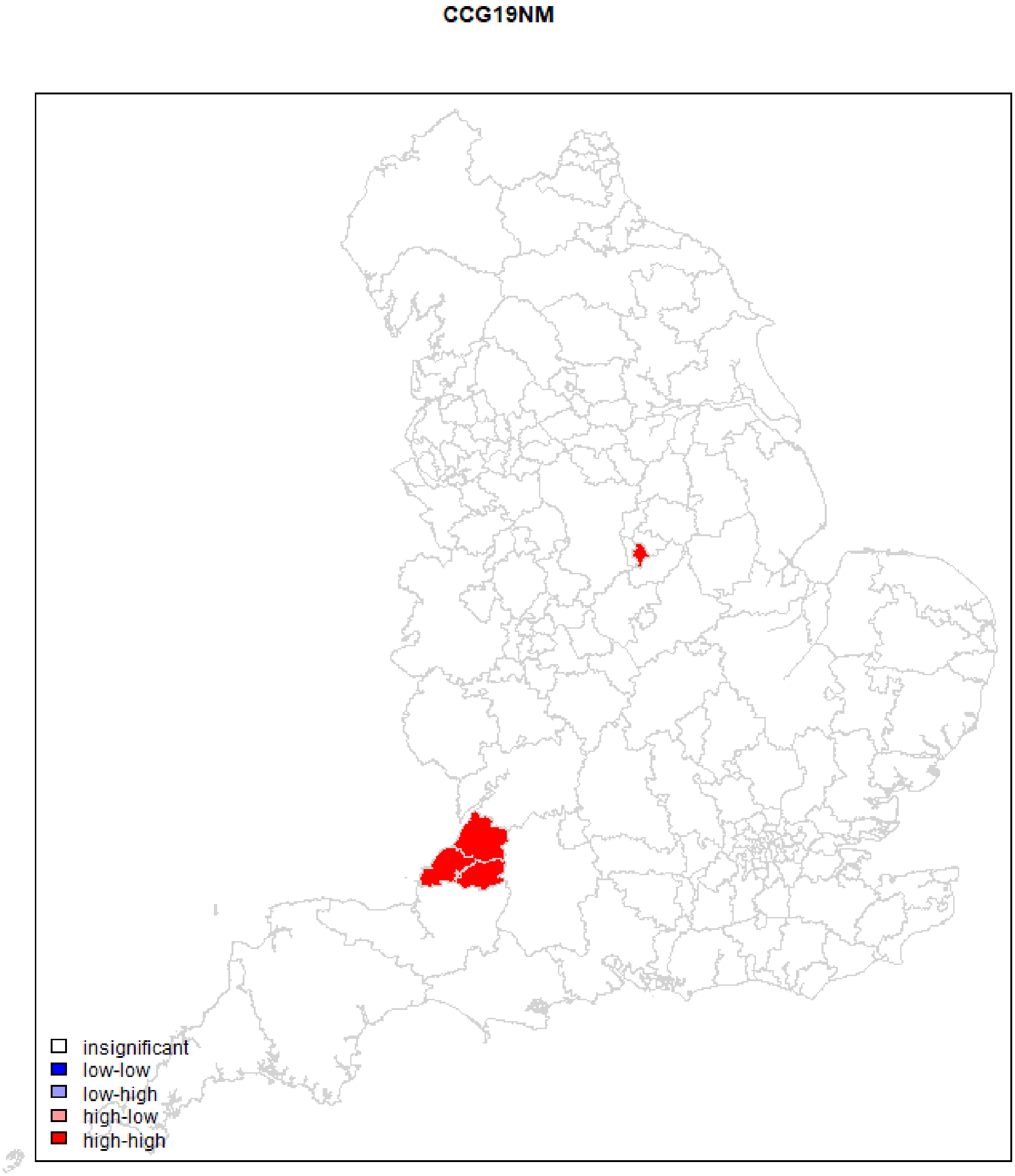

S.18. Zoomed in maps

In case the map in figure 1 is difficult to see some of the city regions in England, below we present zoomed in maps for a) the North West of England, b) the West Midlands, c) London.

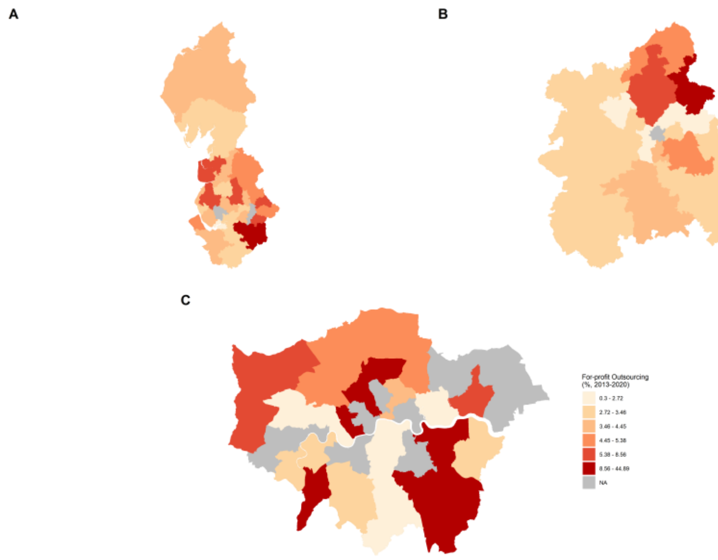

S.19. Ethnicity breakdown

In the main findings we control for ethnic minority % but there is some available data for further breakdowns in ethnicity. Due to data suppression and small numbers, there is lots of missing data and our observations are reduced by over half. Nonetheless we present these breakdowns below. Our main finding holds in all instances.

	ln(T. Mortality)			
	**(1)**	**(2)**	**(3)**	**(4)**
For-profit Outsourcing (%)	0.0053*	0.0056**	0.0054*	0.0056**
	(0.0020)	(0.0019)	(0.0019)	(0.0019)
	[0.0012, 0.0095]	[0.0015, 0.0097]	[0.0013, 0.0095]	[0.0015, 0.0097]
Black/Black British (%)	0.0007			
	(0.0038)			
	[-0.0077, 0.0090]			
Mixed ethnicity (%)		0.0150+		
		(0.0075)		
		[-0.0026, 0.0326]		
Indian (%)			0.0034	
			(0.0042)	
			[-0.0062, 0.0130]	
Bangladeshi/ Pakistani (%)				-0.0072+
				(0.0038)
				[-0.0155, 0.0012]
Num.Obs.	290	290	290	290
R2	0.922	0.923	0.922	0.923
R2 Adj.	0.885	0.886	0.885	0.886
AIC	-669.1	-673.2	-669.8	-673.2
BIC	-316.8	-320.9	-317.4	-320.9
RMSE	0.05	0.05	0.05	0.05

+ p < 0.1, * p < 0.05, ** p < 0.01, *** p < 0.001

Data

S.20. Data Cleaning

For the statistical analysis in this paper, data is aggregated annually. This means constructing an annual variable of for-profit outsourcing. The data for this variable starts in April 2013 and ends in March 2020. Consequently, we use a % of spend going to for-profit companies rather than an absolute value – which would vary a lot from 2013-2014 and 2019-2020.

When aggregating CCG mortality and population data, we use CCG19CD codes to avoid matching on names. This is important given the many mergers to CCGs taking place in 2020. For non-mortality data used at the CCG scale (income, LA spending, occupation, ethnicity, unemployment rate, claimant rate and education rates) this data is only available at the LA level, consequently we take averages of overlapping LAs for each CCG. An ONS best-fit lookup is used for this purpose.

For the MLM we use LA-level mortality data. LA-level mortality rates are only available as rolling 3-year periods. Consequently, we construct expenditure variables for 3-year periods. For the control variables we calculate a 3-year mean for each LA.

S.21. Missing Data

There are two limitations from missing data in this paper. The first is the missing data in the expenditure data. There are many instances where CCGs did not report their expenditure on their websites, or the format was not machine readable. While there is a chance that not reporting of data is symbolic of poor-quality healthcare management and therefore not random. However, it seems unlikely that outsourcing and likelihood of reporting data is associated and therefore we would not expect our finding to impacted by this. We also conduct many sensitivity checks removing or altering the data slightly and always find robust results.

The second issue with missing data was created by the large number of CCG mergers in 2020. Although our expenditure data is not affected by this as it was collected up until this moment, the mortality data for 2019 and 2020, released after the mergers, is not available for CCGs. There is a chance that the mergers happened non-randomly to treatable mortality rates. However the results all hold when only using data before 2019, so we have taken care to account for this bias.

S.21.1 Missing Data of CCG Expenditure and Mortality

To show the entire extent of the available data, and therefore missing data too, below we plot which years we have data available for in each of the CCGs included in this analysis for the two key variables.

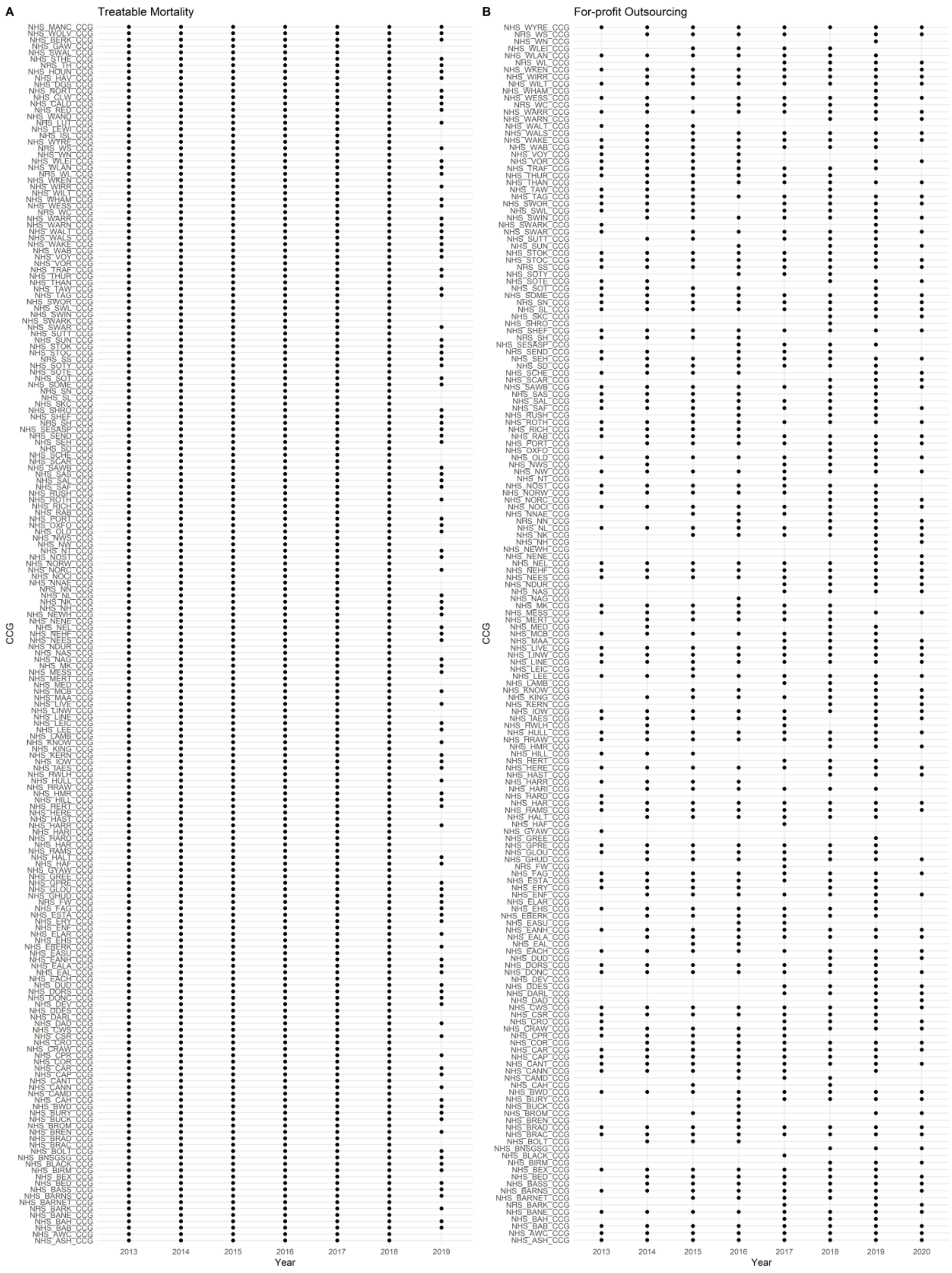

S.22. Model Data Summary

Below we present summaries of all variables included in all the statistical models. Summaries here are for complete observations included in the analysis.

	Mean (SD)	Median (IQR)	Source
Treatable Mortality Rate	85.87 (0.62)	83.7 (22.25)	ONS
For-Profit Outsourcing (%)	5.76 (0.38)	4.04 (3.41)	Rahal & Mohan, (2022).[19]
Total CCG Spend (£Ms)	24.3 (0.75)	19.1 (20.48)	Rahal & Mohan, (2022).[19]
Local Authority Spend (per Capita)	1.27 (0.05)	1.5 (1.61)	MHCLG (RSX)
Claimant Rate	2.07 (0.04)	1.83 (1.39)	ONS (Claimant Count)
Population size	285514.3 (6141.27)	239855 (149055)	ONS
Unemployment Rate	5.36 (0.08)	4.9 (2.6)	ONS (APS)
Ethnic Minority (%)	10.31 (0.4)	5.8 (10.3)	ONS (APS)
Degree Education (%)	35.49 (0.34)	34.3 (11.22)	ONS (APS)
Average Disposable H.hold Income	19952.09 (275.28)	18774 (4641)	ONS (GDHI)
Managerial or Professional Occupation (%)	30.34 (0.23)	29.8 (8.3)	ONS (APS)

S.23. Data Locations

Below we present a table with the full location of data used to create all variables in this analysis. And a brief discussion about the strengths and weaknesses of each.

Variable	Source	Data Location	Strengths	Weaknesses
Treatable Mortality Rate	ONS	https://www.ons.gov.uk/peoplepopulationandcommunity/healthandsocialcare/causesofdeath/datasets/avoidablemortalitybyclinicalcommissioninggroupsinenglandandhealthboardsinwales	Treatable mortality is a useful measure because it is a measure of health outcome which can a) be measured at a population level and b) only capture outcomes which can be improved by the health system – rather than broader health measures such as life expectancy	CCG reforms in 2020 meant that 2019 data was reported with new merged CCGs so we lose a few observations in that year. Due to the small numbers we cannot break down treatable mortality into specific causes of death very well. For instance a useful analysis could be to see how outsourcing relates with mortality from medical accidents but this is not possible due to data suppression.
For-profit Outsourcing (%)	Rahal, C., & Mohan, J. (2022, January 27). The Role of the Third Sector in Public Health Service Provision: Evidence from 25,338 heterogeneous procurement datasets. https://doi.org/10.31235/osf.io/t4x52	https://doi.org/10.5281/zenodo.5054679	The strengths of this variable are the precision of being able to estimate the exact percent of reported expenditure which goes to for profit companies. Previously, the 'healthcare from non-NHS organisations' category of the CCG accounts would have to be used but the data underlying these numbers was not published.	Lack of reporting by some CCGs means that the data is not complete. Some error may exist in the data from the process of matching supplier names to companies house register. The services for which the payments are made are not reported.
Total CCG Spend (£Ms)	Rahal, C., & Mohan, J. (2022, January 27). The Role of the Third Sector in Public Health Service Provision: Evidence from 25,338 heterogeneous procurement datasets. https://doi.org/10.31235/osf.io/t4x52	https://doi.org/10.5281/zenodo.5054679	A specific measure which legally needs to report all expenditure by CCGs over £25,000. By being able to calculate the total expenditure by a given CCG we can control for any confounding of total healthcare service provided and the percentage of that delivered by the private sector.	Lack of reporting by some CCGs means that the data is not complete.
Local Authority Spend (per Capita)	MHCLG (RSX)	https://www.gov.uk/government/collections/local-authority-revenue-expenditure-and-financing	Total service expenditure includes services such as social care, public health and environmental services. This is complete data at the LA level which	This does not give as a detailed insight into the role of joint commissioning or how LAs influence CCG procurement of nursing

			allows us to control for any confounding from local authority expenditure – which over this period suffered from large funding cuts.	homes, some of which will be owned and run by LAs.
Claimant Rate	ONS (Claimant Count)	https://www.nomisweb.co.uk/query/select/getdatasetbytheme.asp?opt=3&theme=&subgrp=	Claimant rate is a good measure for being able to calculate the labour market activity.	Data is reported at the LA level so we aggregate a mean based on overlapping geographies.
Population size	ONS	https://www.ons.gov.uk/peoplepopulationandcommunity/populationandmigration/populationestimates/datasets/clinicalcommissioninggroupmidyearpopulationestimates	Population size is important given changes to the size of a population are often described as being difficult for health systems – particularly given underfunding for these changes. Given the panel data analysis we use, changes in population also represent changes to population density.	We analyse within CCGs but there is more to be assessed in terms of whether rural and urban areas experience this phenomenon differently. The overall population variable cannot help us understand this.
Unemployment Rate	ONS (APS)	https://www.nomisweb.co.uk/query/select/getdatasetbytheme.asp?opt=3&theme=&subgrp=	There will be some overlap between unemployment and claimant count but unemployment will only measure those without any job so covers a more acute definition of labour market activity.	Data is reported at the LA level so we aggregate a mean based on overlapping geographies.
Ethnic Minority (%)	ONS (APS)	https://www.nomisweb.co.uk/query/select/getdatasetbytheme.asp?opt=3&theme=&subgrp=	It is important to control for changes in ethnicity of populations given the disproportionate impact of poor health care on ethnic minorities.	This is a blunt measure of the % of ethnic minority and does not break down different ethnicities. While such an analysis is beyond the remit of this paper, it is an important question worth exploring.
Degree Education (%)	ONS (APS)	https://www.nomisweb.co.uk/query/select/getdatasetbytheme.asp?opt=3&theme=&subgrp=	This measures the percent of people holding degree-level qualifications in a population.	Data is reported at the LA level so we aggregate a mean based on overlapping geographies.
Average Disposable H.hold Income	ONS (GDHI)	https://www.ons.gov.uk/economy/regionalaccounts/grossdisposablehouseholdincome/datasets/regionalgrossdisposablehouseholdincomelocalauthoritiesbyitl1region	This is a good measure of income – which will be important in terms of ability to access alternative healthcare in the case of deteriorating healthcare quality from the NHS	This is a blunt measure of the average income and cannot assess the % of very wealthy people able to afford regular private care nor the % of people experiencing extreme poverty.
Managerial or Professional Occupation (%)	ONS (APS)	https://www.nomisweb.co.uk/query/select/getdatasetbytheme.asp?opt=3&theme=&subgrp=	This measures the percent of people with managerial or professional occupations, which tells us something about the occupational class of an area. It may also control for the % of people with a possibility of receiving some form of private health insurance from their employer.	This cannot break down the impact of healthcare outsourcing/ deterioration on the health of different social classes.

S.24. Causes of Treatable Mortality

Treatable mortality rates are a measure of health system performance based on the number of deaths from causes considered to be avoidable through direct medical intervention. Below we present all the causes that are counted in this measure. Some are marked 50% because they are considered 50% treatable (medical intervention) and 50% preventable (public health intervention).

Condition group and cause	ICD-10 codes	Age	Treatable
**Infectious diseases**			
Tuberculosis	A15-A19, B90, J65	0-74	• (50%)
Scarlet fever	A38	0-74	•
Sepsis	A40 (excl. A40.3), A41 (excl. A41.3)	0-74	•
Cellulitis	A46, L03	0-74	•
Legionnaires disease	A48.1	0-74	•
Streptococcal and enterococci infection	A49.1	0-74	•
Other meningitis	G00.2, G00.3, G00.8, G00.9	0-74	•
Meningitis due to other and unspecified causes	G03	0-74	•
**Neoplasms**			
Cervical cancer	C53	0-74	• (50%)
Colorectal cancer	C18-C21	0-74	•
Breast cancer (female only)	C50	0-74	•
Uterus cancer	C54, C55	0-74	•
Testicular cancer	C62	0-74	•
Thyroid cancer	C73	0-74	•
Hodgkin's disease	C81	0-74	•
Lymphoid leukaemia	C91.0, C91.1	0-74	•
Benign neoplasm	D10-D36	0-74	•
**Endocrine and metabolic diseases**			
Diabetes mellitus	E10-E14	0-74	• (50%)
Thyroid disorders	E00-E07	0-74	•
Adrenal disorders	E24-E25 (excl. E24.4), E27	0-74	•
**Diseases of the nervous system**			
Epilepsy	G40, G41	0-74	•
**Diseases of the circulatory system**			
Aortic aneurysm	I71	0-74	• (50%)
Hypertensive diseases	I10-I13, I15	0-74	• (50%)
Ischaemic heart diseases	I20-I25	0-74	• (50%)
Cerebrovascular diseases	I60-I69	0-74	• (50%)
Other atherosclerosis	I70, I73.9	0-74	• (50%)
Rheumatic and other heart diseases	I00-I09	0-74	•
Venous thromboembolism	I26, I80, I82.9	0-74	•
**Diseases of the respiratory system**			
Upper respiratory infections	J00-J06, J30-J39	0-74	•
Pneumonia, not elsewhere classified or organism unspecified	J12, J15, J16-J18	0-74	•

Acute lower respiratory infections	J20-J22	0-74	•
Asthma and bronchiectasis	J45-J47	0-74	•
Adult respiratory distress syndrome	J80	0-74	•
Pulmonary oedema	J81	0-74	•
Abscess of lung and mediastinum pyothorax	J85, J86	0-74	•
Other pleural disorders	J90, J93, J94	0-74	•
**Diseases of the digestive system**			
Gastric and duodenal ulcer	K25-K28	0-74	•
Appendicitis	K35-K38	0-74	•
Abdominal hernia	K40-K46	0-74	•
Cholelithiasis and cholecystitis	K80-K81	0-74	•
Other diseases of gallbladder or biliary tract	K82-K83	0-74	•
Acute pancreatitis	K85.0, K85.1, K85.3, K85.8, K85.9	0-74	•
Other diseases of pancreas	K86.1, K86.2, K86.3, K86.8, K86.9	0-74	•
**Diseases of the genitourinary system**			
Nephritis and nephrosis	N00-N07	0-74	•
Obstructive uropathy	N13, N20-N21, N35	0-74	•
Renal failure	N17-N19	0-74	•
Renal colic	N23	0-74	•
Disorders resulting from renal tubular dysfunction	N25	0-74	•
Unspecified contracted kidney, small kidney of unknown cause	N26-N27	0-74	•
Inflammatory diseases of genitourinary system	N34.1, N70-N73, N75.0, N75.1, N76.4, N76.6	0-74	•
Prostatic hyperplasia	N40	0-74	•
**Pregnancy, childbirth and the perinatal period**			
Pregnancy, childbirth and the puerperium	O00-O99	0-74	•
Certain conditions originating in the perinatal period	P00-P96	0-74	•
**Congenital malformations**			
Congenital malformations of the circulatory system (heart defects)	Q20-Q28	0-74	•
**Adverse effects of medical and surgical care**			
Drugs, medicaments and biological substances causing adverse effects in therapeutic use	Y40-Y59	0-74	•
Misadventures to patients during surgical and medical care	Y60-Y69, Y83-Y84	0-74	•
Medical devices associated with adverse incidents in diagnostic and therapeutic use	Y70–Y82	0-74	•

S.25. CCG Level data

S.25.1 Table of Outsourcing and Treatable Mortality

Below we present a table with the total levels of Outsourcing for each CCG as well as the average treatable mortality rate for each CCG and the total number of treatable deaths between 2013 and 2020.

	CCG	Total Spend (£10Ms)	Private sector spend (£10Ms)	For-profit outsourcing (%)	Total Treatable Deaths	Average Treatable Mortality Rate
1	NHS_ASH_CCG	89.1967	4.1937	4.7017	479.5	70.7500
2	NHS_AWC_CCG	121.4167	2.4123	1.9868	688.5	74.9833
3	NHS_BAB_CCG	138.4894	5.8414	4.2180	1380.0	78.3857
4	NHS_BAH_CCG	58.8687	3.7538	6.3765	1298.0	83.9857
5	NHS_BANE_CCG	121.2854	14.1856	11.6961	630.5	67.6667
6	NHS_BARK_CCG	11.6919	0.7148	6.1133	908.5	113.7714
7	NHS_BARNET_CCG	134.5444	6.0226	4.4763	1056.0	65.9833
8	NHS_BARNS_CCG	213.3432	5.2981	2.4834	1682.0	94.8000
9	NHS_BASS_CCG	63.3614	3.6676	5.7884	812.5	88.3143
10	NHS_BED_CCG	274.7652	25.5960	9.3156	2105.5	78.6143
11	NHS_BEX_CCG	106.4552	3.0015	2.8195	882.5	78.4167
12	NHS_BIRM_CCG	286.9020	14.6588	5.1093	6754.5	99.6571
13	NHS_BLACK_CCG	1.9550	0.1673	8.5573	1415.5	133.9571
14	NHS_BNSGSG_CCG	235.9059	42.2482	17.9089	4665.5	78.5286
15	NHS_BOLT_CCG	61.7357	4.2177	6.8318	2034.5	105.6571
16	NHS_BRAC_CCG	75.8158	2.1120	2.7857	359.0	161.8333
17	NHS_BRAD_CCG	250.4083	6.8274	2.7265	1659.0	109.5500
18	NHS_BREN_CCG	3.0066	0.1443	4.7993	1241.0	83.4571
19	NHS_BROM_CCG	31.8450	3.8672	12.1437	1060.0	66.5000
20	NHS_BUCK_CCG	7.7327	0.1529	1.9778	2378.0	63.3857
21	NHS_BURY_CCG	72.9005	3.0352	4.1635	1212.5	92.9143
22	NHS_BWD_CCG	127.7320	8.3127	6.5079	1032.5	115.1000
23	NHS_CAH_CCG	5.0151	0.1782	3.5525	1027.0	104.8000
24	NHS_CAMD_CCG	4.8493	0.5652	11.6563	626.5	67.9667
25	NHS_CANN_CCG	44.5648	0.8544	1.9171	849.5	82.4000
26	NHS_CANT_CCG	157.9543	8.3393	5.2796	852.5	76.1500
27	NHS_CAP_CCG	504.6382	20.2925	4.0212	4385.5	74.9286
28	NHS_CAR_CCG	323.1070	9.4394	2.9215	2123.5	91.3143
29	NHS_COR_CCG	38.9518	1.0503	2.6964	352.0	112.6833
30	NHS_CPR_CCG	72.0828	3.3643	4.6672	1072.0	74.3286
31	NHS_CRAW_CCG	111.1547	2.0091	1.8075	402.5	87.5500
32	NHS_CRO_CCG	33.1473	0.8641	2.6069	1442.5	88.5667
33	NHS_CSR_CCG	132.0259	4.5612	3.4548	1105.0	85.1714
34	NHS_CWS_CCG	404.0761	13.2337	3.2751	2289.5	74.4667
35	NHS_DAD_CCG	118.8040	4.7660	4.0116	6451.5	85.7571
36	NHS_DARL_CCG	17.6710	1.1233	6.3565	532.5	92.7667
37	NHS_DDES_CCG	68.6262	4.1470	6.0429	1526.0	96.6333
38	NHS_DEV_CCG	13.0000	0.4656	3.5814	7181.5	76.4143
39	NHS_DONC_CCG	194.8222	16.0047	8.2151	2134.5	96.9000
40	NHS_DORS_CCG	564.0929	24.4047	4.3264	4424.5	71.3571
41	NHS_DUD_CCG	77.0217	3.3675	4.3722	1789.0	88.9714
42	NHS_EACH_CCG	28.5459	10.4597	36.6417	825.5	69.3667
43	NHS_EAL_CCG	31.0159	0.7564	2.4389	1400.5	86.8000
44	NHS_EALA_CCG	263.6262	13.7482	5.2150	2769.0	101.7857
45	NHS_EANH_CCG	411.3464	16.7221	4.0652	2730.0	75.8429
46	NHS_EASU_CCG	27.5225	2.6953	9.7931	675.0	73.5000
47	NHS_EBERK_CCG	236.6322	4.5800	1.9355	1668.0	76.6714
48	NHS_EHS_CCG	126.5896	3.0757	2.4296	878.5	76.5333
49	NHS_ELAR_CCG	5.6447	0.3613	6.4004	1737.5	67.6000
50	NHS_ENF_CCG	62.6354	2.8353	4.5267	1050.0	77.8667
51	NHS_ERY_CCG	166.8333	12.6663	7.5922	2212.5	79.8714
52	NHS_ESTA_CCG	59.9979	8.1278	13.5468	810.0	87.7429
53	NHS_FAG_CCG	147.5144	4.7309	3.2071	977.0	73.4857
54	NHS_FW_CCG	1.9550	0.1673	8.5573	1486.5	91.0857
55	NHS_GHUD_CCG	148.7779	18.6483	12.5343	1312.5	88.8286
56	NHS_GLOU_CCG	451.8417	13.3179	2.9475	3547.0	75.1857
57	NHS_GPRE_CCG	151.9930	5.9471	3.9127	1262.0	95.9857
58	NHS_GREE_CCG	3.0216	0.3164	10.4725	950.0	94.8500
59	NHS_GYAW_CCG	1.7797	0.2068	11.6217	1159.0	87.1833
60	NHS_HAF_CCG	3.4430	0.0688	1.9983	626.5	81.6143
61	NHS_HALT_CCG	94.9133	1.6843	1.7746	960.0	103.1286
62	NHS_HAMS_CCG	247.3962	16.6362	6.7245	802.5	63.9333
63	NHS_HAR_CCG	144.5336	6.7353	4.6600	1073.5	93.2000
64	NHS_HARD_CCG	1.9596	0.0067	0.3434	642.5	68.8333
65	NHS_HARI_CCG	13.5318	1.9170	14.1665	839.5	89.2167
66	NHS_HARR_CCG	44.8492	2.4334	5.4258	896.0	68.5143
67	NHS_HAST_CCG	38.6745	0.8367	2.1633	1436.0	96.5667
68	NHS_HERE_CCG	142.1713	4.3873	3.0859	907.0	78.1333

69	NHS_HERT_CCG	186.4982	10.0641	5.3964	2745.0	73.3857
70	NHS_HILL_CCG	57.0294	3.2323	5.6678	1163.5	81.9000
71	NHS_HMR_CCG	53.0589	2.4846	4.6827	1577.0	112.2286
72	NHS_HRAW_CCG	90.5708	1.4612	1.6133	663.0	68.9500
73	NHS_HULL_CCG	199.7943	36.7281	18.3830	1874.0	117.9286
74	NHS_HWLH_CCG	22.6649	4.2653	18.8189	668.0	63.9667
75	NHS_IAES_CCG	137.5530	11.7194	8.5199	2257.5	72.3000
76	NHS_IOW_CCG	87.2929	1.9513	2.2353	856.0	77.5286
77	NHS_KERN_CCG	126.6256	7.9390	6.2697	3685.0	78.1429
78	NHS_KING_CCG	74.0873	12.1660	16.4212	489.0	68.7833
79	NHS_KNOW_CCG	26.2597	1.0881	4.1438	1157.0	115.7286
80	NHS_LAMB_CCG	42.5439	0.5722	1.3449	955.0	99.7667
81	NHS_LEE_CCG	609.7731	27.3572	4.4865	4338.5	93.6571
82	NHS_LEIC_CCG	2.8155	0.1556	5.5255	1948.0	110.5286
83	NHS_LINE_CCG	150.5100	4.7180	3.1347	1516.5	97.3167
84	NHS_LINW_CCG	161.2271	7.6162	4.7239	1166.0	90.8667
85	NHS_LIVE_CCG	422.9544	18.4691	4.3667	3273.0	113.6000
86	NHS_MAA_CCG	39.4745	2.4474	6.1998	1078.5	99.2333
87	NHS_MCB_CCG	184.0824	5.8044	3.1532	2124.5	80.9857
88	NHS_MED_CCG	25.6557	4.9932	19.4624	1212.5	91.1333
89	NHS_MERT_CCG	72.9919	2.1740	2.9784	633.5	78.3333
90	NHS_MESS_CCG	238.9167	25.0644	10.4909	2029.0	70.1714
91	NHS_MK_CCG	81.7869	3.9037	4.7730	1184.5	83.1000
92	NHS_NAG_CCG	11.1884	0.2961	2.6461	2967.0	96.9714
93	NHS_NAS_CCG	28.1428	1.4429	5.1269	540.0	76.3000
94	NHS_NDUR_CCG	48.5737	1.9554	4.0255	1122.5	82.3500
95	NHS_NEES_CCG	258.6982	32.5699	12.5899	2133.5	83.8000
96	NHS_NEHF_CCG	153.1753	9.6364	6.2911	815.0	66.9143
97	NHS_NEL_CCG	137.1784	26.5189	19.3317	1079.0	94.0000
98	NHS_NENE_CCG	43.9367	2.7153	6.1800	2904.0	85.5333
99	NHS_NEWH_CCG	2.3938	0.0071	0.2986	1173.5	100.6571
100	NHS_NH_CCG	3.9765	0.2041	5.1329	922.5	67.5000
101	NHS_NK_CCG	101.5054	13.0264	12.8332	1035.5	96.0571
102	NHS_NL_CCG	106.1363	3.4217	3.2238	1184.0	90.6429
103	NHS_NN_CCG	37.0446	1.3956	3.7673	861.0	71.8167
104	NHS_NNAE_CCG	73.3396	6.4838	8.8408	660.0	79.7500
105	NHS_NOCI_CCG	225.8444	45.3639	20.0863	1375.5	118.3333
106	NHS_NORC_CCG	46.6250	1.6254	3.4861	2187.5	84.7286
107	NHS_NORW_CCG	118.1798	4.4186	3.7389	823.0	80.5500
108	NHS_NOST_CCG	94.2442	5.0527	5.3613	1438.0	84.0571
109	NHS_NT_CCG	2.0774	0.0344	1.6555	1345.0	89.0000
110	NHS_NW_CCG	69.4064	6.6690	9.6086	486.5	77.6667
111	NHS_NWS_CCG	45.7581	5.4882	11.9940	1233.0	72.1833
112	NHS_OLD_CCG	117.5544	9.4533	8.0417	1649.5	109.7286
113	NHS_OXFO_CCG	74.1177	1.9666	2.6533	3030.5	68.6429
114	NHS_PORT_CCG	148.6379	7.0648	4.7530	1113.5	95.4714
115	NHS_RAB_CCG	122.6959	3.5011	2.8535	822.5	81.3833
116	NHS_RICH_CCG	41.7409	1.1773	2.8206	563.0	61.8333
117	NHS_ROTH_CCG	199.6942	5.3701	2.6892	1954.0	100.5143
118	NHS_RUSH_CCG	52.5452	6.1027	11.6143	446.5	69.6667
119	NHS_SAF_CCG	106.4715	4.1606	3.9077	743.0	76.9000
120	NHS_SAL_CCG	222.7918	7.0117	3.1472	1669.5	113.7000
121	NHS_SAS_CCG	52.8758	3.2265	6.1021	898.0	74.1286
122	NHS_SAWB_CCG	390.6946	19.8621	5.0838	2705.5	116.5857
123	NHS_SCAR_CCG	31.3256	1.6070	5.1300	640.0	90.1500
124	NHS_SCHE_CCG	57.8572	1.9774	3.4178	905.5	88.1167
125	NHS_SD_CCG	164.7748	20.6561	12.5360	968.5	61.9667
126	NHS_SEH_CCG	158.5507	6.7024	4.2273	1033.5	70.2286
127	NHS_SEND_CCG	53.3497	2.6433	4.9548	1111.5	88.7000
128	NHS_SESASP_CCG	9.7573	0.2299	2.3563	1377.0	77.1714
129	NHS_SH_CCG	32.5019	0.4297	1.3221	344.5	57.9000
130	NHS_SHEF_CCG	442.5339	16.3329	3.6908	3007.0	87.2714
131	NHS_SHRO_CCG	3.1941	0.1066	3.3369	1736.5	76.0143
132	NHS_SKC_CCG	53.4247	4.2064	7.8734	1055.5	84.6000
133	NHS_SL_CCG	87.6896	2.8980	3.3048	698.0	78.5167
134	NHS_SN_CCG	131.6253	5.8571	4.4498	944.5	67.8000
135	NHS_SOME_CCG	406.1872	19.6904	4.8476	3169.5	69.9714
136	NHS_SOT_CCG	69.1321	3.3099	4.7878	1049.0	91.5000
137	NHS_SOTE_CCG	59.3278	1.7189	2.8973	1645.0	113.2000
138	NHS_SOTY_CCG	9.8111	0.1832	1.8677	1045.0	93.0000
139	NHS_SS_CCG	140.6365	3.9594	2.8154	1184.0	99.3143
140	NHS_STOC_CCG	232.4979	13.6225	5.8592	1688.5	80.5000
141	NHS_STOK_CCG	117.8769	8.4045	7.1299	1750.5	98.3000
142	NHS_SUN_CCG	61.9920	2.6068	4.2051	1985.0	97.2571
143	NHS_SUTT_CCG	69.7308	1.9908	2.8550	675.0	73.8000
144	NHS_SWAR_CCG	96.1228	3.0818	3.2061	1274.0	72.6429
145	NHS_SWARK_CCG	12.2270	0.1375	1.1250	908.0	92.7167
146	NHS_SWIN_CCG	116.8057	6.0906	5.2143	915.5	86.5000
147	NHS_SWL_CCG	74.3440	2.7406	3.6863	623.5	83.1833
148	NHS_SWOR_CCG	77.5220	2.9516	3.8075	1340.0	75.8333

149	NHS_TAG_CCG	165.9142	7.6193	4.5923	1868.0	103.6286
150	NHS_TAW_CCG	46.0825	0.5581	1.2112	974.5	94.4714
151	NHS_THAN_CCG	67.7331	2.1346	3.1515	802.0	96.6500
152	NHS_THUR_CCG	77.9838	2.0130	2.5814	858.5	86.9000
153	NHS_TRAF_CCG	149.6637	6.6295	4.4296	1200.5	79.4571
154	NHS_VOR_CCG	33.1918	0.9497	2.8612	478.0	81.9333
155	NHS_VOY_CCG	126.6822	1.2983	1.0249	1988.5	77.7286
156	NHS_WAB_CCG	202.8905	7.1108	3.5048	2443.0	104.2714
157	NHS_WAKE_CCG	273.4190	13.4785	4.9296	2416.5	96.9571
158	NHS_WALS_CCG	177.3103	5.5416	3.1254	1650.0	103.5000
159	NHS_WALT_CCG	53.8998	2.5473	4.7260	1090.5	93.7714
160	NHS_WARN_CCG	24.5900	0.7369	2.9968	1140.0	90.8429
161	NHS_WARR_CCG	122.9043	3.5833	2.9155	1302.0	88.3714
162	NHS_WC_CCG	37.7062	1.3124	3.4807	999.0	76.2667
163	NHS_WESS_CCG	223.3604	7.6282	3.4152	1527.5	73.4714
164	NHS_WHAM_CCG	6.0896	0.3332	5.4709	2370.5	61.8286
165	NHS_WILT_CCG	326.7789	39.7173	12.1542	1944.5	70.4833
166	NHS_WIRR_CCG	260.6889	12.3664	4.7437	2306.5	93.3857
167	NHS_WKEN_CCG	308.6818	11.9971	3.8866	1800.0	71.3667
168	NHS_WL_CCG	10.7974	4.8475	44.8950	705.0	61.5571
169	NHS_WLAN_CCG	77.8140	5.1574	6.6278	725.5	82.8857
170	NHS_WLEI_CCG	35.4466	1.6765	4.7297	2148.5	73.8429
171	NHS_WN_CCG	11.8710	0.5032	4.2389	866.0	77.7667
172	NHS_WS_CCG	49.7483	4.1690	8.3803	1125.0	65.6429
173	NHS_WYRE_CCG	61.3737	2.6873	4.3787	515.0	81.5000

S.25.2 Stripe plot

We can also present the monthly changes to outsourcing for each CCG. Here we limit the scale to 0-10 to be able to show the variation between CCGs and across time.

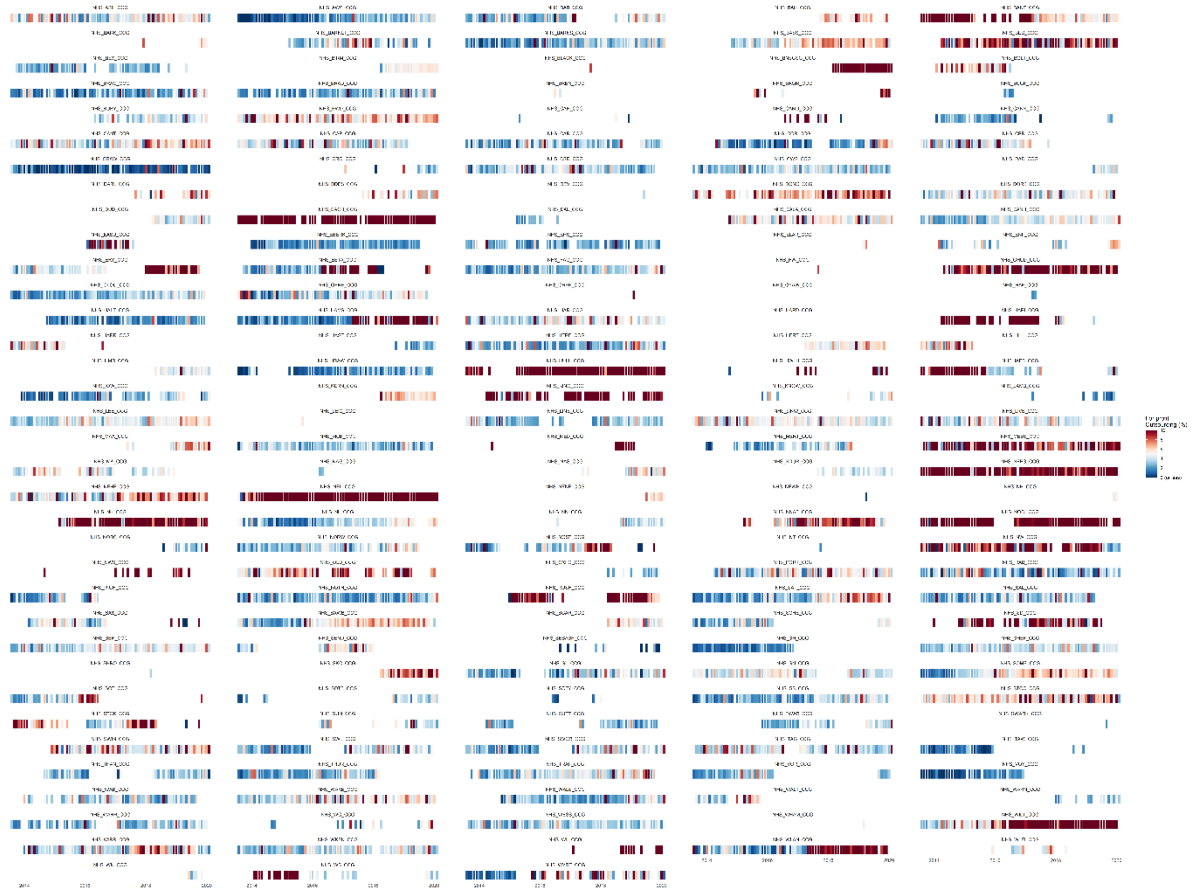

Reporting Checklists and Statements

S.26. STROBE Checklist

	Item No.	Recommendation	Page No.	Relevant text from manuscript
**Title and abstract**	1	(*a*) Indicate the study's design with a commonly used term in the title or the abstract	1	Observational analysis
		(*b*) Provide in the abstract an informative and balanced summary of what was done and what was found	1	"estimate multivariate longitudinal regression models with CCG-level fixed effects analysing the effects of for-profit outsourcing on treatable mortality rates in the following year.“<br>"An annual increase of one percentage point of outsourcing to the private sector corresponds with an annual increase in treatable mortality of 0.38% or 0.29 deaths per 100,000 population (95% CI 0·15% to 0·62%; p= 0.0055) in the following year."
**Introduction**				
Background/rationale	2	Explain the scientific background and rationale for the investigation being reported	3	"But the evidence on the impact of 'creeping privatisation' in general and in England's NHS specifically remains uncertain. In general, these findings are often inconclusive in that they do not analyse the aggregate effect of outsourcing on service-wide performance [13, 14]. Moreover, such comparisons between for-profit and not-for profit providers are often inappropriate because the case-mixes of private and public services are quite different.“
Objectives	3	State specific objectives, including any prespecified hypotheses	4	"In this paper, we examine the impact on treatable mortality of increased outsourcing to private for-profit providers from England's CCGs during the period immediately following the implementation of the 2012 Health and Social Care Act. To do this, we draw on an entirely novel data set which brings together every reported financial transaction between CCGs and private healthcare provides across 173 CCGs. This data allows us to conduct, to our knowledge, the first empirical evaluation of one of the most controversial reforms in England's recent history.“
**Methods**				
Study design	4	Present key elements of study design early in the paper	5	"We ran fixed effects and first differences regression models on the association between outsourcing and treatable mortality, these models will control for all time invariant confounders at the regional level."

Setting	5	Describe the setting, locations, and relevant dates, including periods of recruitment, exposure, follow-up, and data collection	5	"Data was collected on all live English CCGs as of 2019. Of the full 191 sample, 173 provided at least some machine-readable data between 2013 and 2020"
Participants	6	(*a*) *Cohort study*—Give the eligibility criteria, and the sources and methods of selection of participants. Describe methods of follow-up<br><br>*Case-control study*—Give the eligibility criteria, and the sources and methods of case ascertainment and control selection. Give the rationale for the choice of cases and controls<br><br>*Cross-sectional study*—Give the eligibility criteria, and the sources and methods of selection of participants	5	"Data was collected on all live English CCGs as of 2019. Of the full 191 sample, 173 provided at least some machine-readable data between 2013 and 2020"
		(*b*) *Cohort study*—For matched studies, give matching criteria and number of exposed and unexposed<br><br>*Case-control study*—For matched studies, give matching criteria and the number of controls per case	5	"We also ran our fixed effects model using covariate-balancing with propensity scores based on treatable mortality rates at the beginning of the time-series and the total number of General Practitioners in each CCG."
Variables	7	Clearly define all outcomes, exposures, predictors, potential confounders, and effect modifiers. Give diagnostic criteria, if applicable	4-5	"Table 1 lists our study variables. The response variable is our measure for healthcare quality, 'treatable mortality'. This is defined as: "deaths that can be mainly avoided through timely and effective healthcare interventions, including secondary prevention and treatment" [17]."
Data sources/ measurement	8*	For each variable of interest, give sources of data and details of methods of assessment (measurement). Describe comparability of assessment methods if there is more than one group	*5*	*"*Full locations of the data as well as a discussion of the data limitations is available in table S.22. Data here is for complete observations included in the analysis. For a description and discussion of missing data see S.21. in supplementary material."

Bias	9	Describe any efforts to address potential sources of bias	6	"To account for potential bias in the main result from the choice of covariates in the model, we present a specification curve in the supplementary materials which is combined with the random error loops (figure S.8.)."<br><br>"Finally we run the linear fixed effects regression (Table 2, model 1) 173 times, removing a different individual CCG on each loop (see supplementary material S.5.). This was done to check whether any single CCG was primarily driving our overall result. We find that all regressions return a statistically significant, positive result we can therefore be confident that our result is not considerably biased by any single CCG."
Study size	10	Explain how the study size was arrived at	5	"Data was collected on all live English CCGs as of 2019. Of the full 191 sample, 173 provided at least some machine-readable data between 2013 and 2020"

S.27. GATHER Checklist

Item number	Checklist item	Page No.	Relevant text from manuscript
Objectives and funding			
1	Define the indicator(s), populations (including age, sex, and geographic entities), and time period(s) for which estimates were made.	4-5	“Table 1 lists our study variables.”<br>“Full locations of the data as well as a discussion of the data limitations is available in table S.22. Data here is for complete observations included in the analysis. For a description and discussion of missing data see S.21. in supplementary material.”
2	List the funding sources for the work.	1	“Funding: This work is supported by The Wellcome Trust [221160/Z/20/Z], [220206/Z/20/Z]“
Data inputs			
For all data inputs from multiple sources that are synthesised as part of the study:			
3	Describe how the data were identified and how the data were accessed.	5	“Full locations of the data as well as a discussion of the data limitations is available in table S.22. Data here is for complete observations included in the analysis. For a description and discussion of missing data see S.21. in supplementary material.”
4	Specify the inclusion and exclusion criteria. Identify all ad-hoc exclusions.	5	“Data was collected on all live English CCGs as of 2019. Of the full 191 sample, 173 provided at least some machine-readable data between 2013 and 2020, although most of those have years missing due to mergers or missing periods in data publication (see supplementary material S.16. for full description of missing data).”
5	Provide information about all included data sources and their main characteristics. For each data source used, report reference information or contact name/institution, population represented, data collection method, year(s) of data collection, sex and age range, diagnostic criteria or measurement method, and sample size, as relevant.	5	“Full locations of the data as well as a discussion of the data limitations is available in table S.22. Data here is for complete observations included in the analysis. For a description and discussion of missing data see S.21. in supplementary material.”
6	Identify and describe any categories of input data that have potentially important biases (eg, based on characteristics listed in item 5).	5	“Full locations of the data as well as a discussion of the data limitations is available in table S.22. Data here is for complete observations included in the analysis. For a description and discussion of missing data see S.21. in supplementary material.”
For data inputs that contribute to the analysis but were not synthesised as part of the study:			
7	Describe and give sources for any other data inputs.	5	“Full locations of the data as well as a discussion of the data limitations is available in table S.22. Data here is for complete observations included in the analysis. For a description and discussion of missing data see S.21. in supplementary material.”
For all data inputs:			

8	Provide all data inputs in a file format from which data can be efficiently extracted (eg, a spreadsheet rather than a PDF), including all relevant meta-data listed in item 5. For any data inputs that cannot be shared because of ethical or legal reasons, such as third-party ownership, provide a contact name or the name of the institution that retains the right to the data.	13	"The extensive code library which accompanies this work can be found at https://github.com/BenGoodair/CCG-Outsourcing. The data that support the findings of this study are all publicly available, replication materials all available at https://github.com/BenGoodair/CCG-Outsourcing. Locations of raw data is detailed in supplementary material S.22. CCG expenditure data available from Rahal and Mohan (2022). [16]"
Data analysis			
9	Provide a conceptual overview of the data analysis method. A diagram may be helpful.	5	"We ran fixed effects and first differences regression models on the association between outsourcing and treatable mortality, these models will control for all time invariant confounders at the regional level. We also ran our fixed effects model using covariate-balancing with propensity scores based on treatable mortality rates at the beginning of the time-series and the total number of General Practitioners in each CCG. Covariate balancing is an advanced matching method which can weight values to balance the model, accounting for differences in observations according to their value of a continuous treatment variable, in this case for-profit outsourcing[18]."
10	Provide a detailed description of all steps of the analysis, including mathematical formulae. This description should cover, as relevant, data cleaning, data pre-processing, data adjustments and weighting of data sources, and mathematical or statistical model(s).	8	"For full model expressions see supplementary material (S.2.)"
11	Describe how candidate models were evaluated and how the final model(s) were selected.	6	"To account for potential bias in the main result from the choice of covariates in the model, we present a specification curve in the supplementary materials which is combined with the random error loops (figure S.8.)."
12	Provide the results of an evaluation of model performance, if done, as well as the results of any relevant sensitivity analysis.	6	"To check whether potential error in the contract data influences our inferences, we synthetically replicate the effect of error on our findings. By running the regression results 50,000 times, each time multiplying the outsourcing values by random numbers we simulate how random error may impact the study's findings."
13	Describe methods of calculating uncertainty of the estimates. State which sources of uncertainty were, and were not, accounted for in the uncertainty analysis.	8	"Robust SEs are clustered at individual level and use a bias-reduced linearization estimator (CR2) [19] Satterthwaite degrees of freedom used in MLM"

14	State how analytical or statistical source code used to generate estimates can be accessed.	13	"The extensive code library which accompanies this work can be found at https://github.com/BenGoodair/CCG-Outsourcing. The data that support the findings of this study are all publicly available, replication materials all available at https://github.com/BenGoodair/CCG-Outsourcing. Locations of raw data is detailed in supplementary material S.22. CCG expenditure data available from Rahal and Mohan (2022). [16]"
Results and discussion			
15	Provide published estimates in a file format from which data can be efficiently extracted.	13	"The extensive code library which accompanies this work can be found at https://github.com/BenGoodair/CCG-Outsourcing. The data that support the findings of this study are all publicly available, replication materials all available at https://github.com/BenGoodair/CCG-Outsourcing. Locations of raw data is detailed in supplementary material S.22. CCG expenditure data available from Rahal and Mohan (2022). [16]"
16	Report a quantitative measure of the uncertainty of the estimates (eg, uncertainty intervals).	8	95% Cis reported in table
17	Interpret results in light of existing evidence. If updating a previous set of estimates, describe the reasons for changes in estimates.		No previous estimates of this relationship
18	Discuss limitations of the estimates. Include a discussion of any modelling assumptions or data limitations that affect interpretation of the estimates.	13	"The associational nature of our findings cannot rule out the possibility of residual confounding, consequently our findings should not be interpreted as necessarily evidencing a causal relationship between outsourcing and mortality rates. The expenditure data does not contain information on the specific services provided by the supplier, as such there remains further research needed to distinguish if some acute services are primarily causing the relationship we observe."

S.28. Study protocol and data analysis plan statements

There was no study protocol or data analysis plan for this research. No sensitive data is included in the research and, as such, no ethics approval was sought.
